# Prediction of Preeclampsia and Intrauterine Growth Restriction: Development of Machine Learning Models on a Prospective Cohort

**DOI:** 10.2196/15411

**Published:** 2020-05-18

**Authors:** Herdiantri Sufriyana, Yu-Wei Wu, Emily Chia-Yu Su

**Affiliations:** 1 Graduate Institute of Biomedical Informatics College of Medical Science and Technology Taipei Medical University Taipei Taiwan; 2 Department of Medical Physiology College of Medicine University of Nahdlatul Ulama Surabaya Surabaya Indonesia; 3 Clinical Big Data Research Center Taipei Medical University Hospital Taipei Taiwan; 4 Research Center for Artificial Intelligence in Medicine Taipei Medical University Taipei Taiwan

**Keywords:** preeclampsia, intrauterine growth restriction, machine learning, uterine artery Doppler, sFlt-1/PlGF ratio

## Abstract

**Background:**

Preeclampsia and intrauterine growth restriction are placental dysfunction–related disorders (PDDs) that require a referral decision be made within a certain time period. An appropriate prediction model should be developed for these diseases. However, previous models did not demonstrate robust performances and/or they were developed from datasets with highly imbalanced classes.

**Objective:**

In this study, we developed a predictive model of PDDs by machine learning that uses features at 24-37 weeks’ gestation, including maternal characteristics, uterine artery (UtA) Doppler measures, soluble fms-like tyrosine kinase receptor-1 (sFlt-1), and placental growth factor (PlGF).

**Methods:**

A public dataset was taken from a prospective cohort study that included pregnant women with PDDs (66/95, 69%) and a control group (29/95, 31%). Preliminary selection of features was based on a statistical analysis using SAS 9.4 (SAS Institute). We used Weka (Waikato Environment for Knowledge Analysis) 3.8.3 (The University of Waikato, Hamilton, NZ) to automatically select the best model using its optimization algorithm. We also manually selected the best of 23 white-box models. Models, including those from recent studies, were also compared by interval estimation of evaluation metrics. We used the Matthew correlation coefficient (MCC) as the main metric. It is not overoptimistic to evaluate the performance of a prediction model developed from a dataset with a class imbalance. Repeated 10-fold cross-validation was applied.

**Results:**

The classification via regression model was chosen as the best model. Our model had a robust MCC (.93, 95% CI .87-1.00, vs .64, 95% CI .57-.71) and specificity (100%, 95% CI 100-100, vs 90%, 95% CI 90-90) compared to each metric of the best models from recent studies. The sensitivity of this model was not inferior (95%, 95% CI 91-100, vs 100%, 95% CI 92-100). The area under the receiver operating characteristic curve was also competitive (0.970, 95% CI 0.966-0.974, vs 0.987, 95% CI 0.980-0.994). Features in the best model were maternal weight, BMI, pulsatility index of the UtA, sFlt-1, and PlGF. The most important feature was the sFlt-1/PlGF ratio. This model used an M5P algorithm consisting of a decision tree and four linear models with different thresholds. Our study was also better than the best ones among recent studies in terms of the class balance and the size of the case class (66/95, 69%, vs 27/239, 11.3%).

**Conclusions:**

Our model had a robust predictive performance. It was also developed to deal with the problem of a class imbalance. In the context of clinical management, this model may improve maternal mortality and neonatal morbidity and reduce health care costs.

## Introduction

Preeclampsia and intrauterine growth restriction (IUGR) are called placental dysfunction–related disorders (PDDs). These diseases have similar pathogeneses, biomarkers, and referral consequences [1,2]. However, they have different phenotypes and various correlations among biomarkers [3]. Subtypes of preeclampsia demonstrate heterogeneous gene expressions, yet a multiomics approach delineated no serological biomarkers [4]. These situations may cause difficulties in developing a robust prediction model for these diseases.

Preeclampsia prevalence ranges from 3% to 5% worldwide as a common disease contributing to maternal mortality [5]. The fetus of a pregnant woman with or without preeclampsia may undergo IUGR, which is associated with neonatal morbidity [6,7]. In spite of difficulties in distinguishing between these two diseases, both of them have similar consequences. They require referral to a hospital accompanied by advanced maternal and neonatal care within a certain time period [8]. Being able to predict PDDs would greatly support clinicians in making referral decisions, which should eventually improve both maternal and neonatal outcomes.

Compared to the traditional first-trimester screening, a prediction model is more reliable for women in several countries if it uses predictors in the second or third trimester. In those countries, women have low numbers of first visits in the first trimester [9]. Meanwhile, models for predicting PDDs have been developed mostly for preeclampsia at 11-13 weeks’ gestation. This period is considered the best time window for its prediction and the most effective prevention method [10,11]. Therefore, if using only the first-trimester prediction, pregnant women in those countries lose the chance to undergo early screening of preeclampsia. Although prevention is still not available after the first trimester, the second- or third-trimester prediction will still impart benefits in the context of clinical management [12]. Decision on early delivery, including by cesarean section, was recommended in the cases of deteriorated maternal or fetal condition [13]. Pregnant women who are more likely to develop preeclampsia can achieve benefit by reaching out to hospitals with advanced maternal care within a certain time period if this condition was well predicted. This benefit is still achieved, although risk of preeclampsia is lately identified at the third trimester, particularly before term (ie, <37 weeks’ gestation), in which early delivery will increase prematurity. Even though the babies were delivered at term from pregnant women who have developed IUGR, they still need advanced neonatal care. It is because low birth weight and in-hospital deaths were found to be more prevalent in those babies compared to those delivered from pregnant women without IUGR [14,15]. Nonetheless, previous models did not demonstrate robust predictive performances using features in any trimester and/or they were developed from datasets with highly imbalanced classes [16-27].

Predictive modeling using conventional statistical methods may be difficult for preeclampsia, since there are various correlations among its predictors [3]. As this disease has heterogeneous gene expressions, another possible difficulty is the noisy class of outcomes [4]. Machine learning methods are capable of dealing with such problems [28]. In addition, a common problem with preeclampsia and/or IUGR is a class imbalance, as models were shown to develop overoptimistic predictions [29]. This study attempted to develop a prediction method for PDDs by machine learning that uses features at 24-37 weeks’ gestation, including maternal characteristics, uterine artery (UtA) Doppler measures, soluble fms-like tyrosine kinase receptor-1 (sFlt-1), and placental growth factor (PlGF).

## Methods

### Study Design

We developed a machine learning model and report it based on Guidelines for Developing and Reporting Machine Learning Predictive Models in Biomedical Research [30]. Our study utilized a public dataset from a prospective cohort study based on STROBE (STrengthening the Reporting of OBservational studies in Epidemiology) guidelines [3]. We developed this model to predict a prognosis of pregnancy outcomes. The prediction model should solve a classification task between a control group and a cohort with a PDD, either preeclampsia or IUGR. A referral decision to a hospital with advanced care is a consequence related to an under- or overprediction of these diseases. Eventually, underprediction may increase maternal mortality and neonatal morbidity, while overprediction may increase health care costs as burdens to either patients or health insurance companies. We intended to avoid both of these scenarios. This goal can be considered to have been achieved if the prediction model demonstrates a higher Matthew correlation coefficient (MCC) than those of recent studies. The range of MCCs is from –1 (worst) to 1 (best). This metric can imply trade-off between underprediction (ie, lower sensitivity and higher specificity) and overprediction (ie, higher sensitivity and lower specificity). This trade-off is commonly evaluated by area under the receiver operating characteristic (ROC) curve (AUC) and accuracy. However, these metrics cannot fairly imply predictive performance in datasets with imbalanced classes [29], like preeclampsia and IUGR. For example, in a low-prevalence event (ie, 10/100, 10%), the predictive performances are still high in terms of sensitivity (ie, 9/10, 90%) and specificity (ie, 81/90, 90%) as parts of AUC. The accuracy (ie, 90/100, 90%) is also still high, but the MCC is not (ie, .62).

### Data Source

The dataset used in this study is a public dataset in the Mendeley Data repository [31]. This dataset belongs to a study conducted at the University Medical Centre Ljubljana, Slovenia [3]. It was approved by the Republic of Slovenia National Medical Ethics Committee (No. 104/04/12). The original study collected data from September 2012 to January 2015. We downloaded this public dataset on March 11, 2019. Inclusion criteria were ≥24 weeks’ gestation at the time of data collection and similar proportions of <34 or ≥34 weeks’ gestation at delivery between the PDD and control groups. For all women with a PDD, the time interval was 48 hours at maximum for the gestational age between data collection and delivery. Exclusion criteria were signs of prepregnancy hypertension, prepregnancy diabetes, hypertensive disorders during pregnancy, or gestational diabetes.

This dataset provides features (ie, predictors) consisting of maternal age (years), parity (nulliparous vs parous), maternal weight before pregnancy (kg), maternal height (m), BMI before pregnancy (kg/m^2^), UtA Doppler measures, sFlt-1 (µg/L), PlGF (µg/L), and the sFlt-1/PlGF ratio. The UtA Doppler measures included the resistivity index (RI) of the UtA (RI-UtA), pulsatility index (PI) of the UtA (PI-UtA), and peak systolic velocity of the UtA (PSV-UtA). Each measure was taken for both the right and left UtAs. The average of both UtAs was calculated. In addition, the presence or absence of a bilateral notch was also included. The class (ie, outcome) consisted of 29 control subjects and 66 women with PDDs: 32 (48%) with both preeclampsia and IUGR, 12 (18%) with IUGR without preeclampsia, and 22 (33%) with preeclampsia without IUGR. Therefore, the ratio of positive (ie, PDD) to negative (ie, control) classes was 7:3. Detailed criteria for the ultrasound examination, blood sampling, and diagnosis of either preeclampsia or IUGR were previously described [3].

There were missing values in one subject for maternal weight, height, and BMI. However, the BMI classification was inferred from the report for that subject (ie, overweight) [3]. Considering the distribution of BMI before pregnancy, a feature was added by discretization (<25 kg/m^2^ [underweight + normal] vs ≥25 kg/m^2^ [overweight + obese]).

### Feature Selection

We used SAS 9.4 (SAS Institute) to conduct preliminary statistical analyses. These intended to identify the relevancy of candidate features by their association with the class. The dataset with relevant features was initially used for comparison with machine learning models. To improve their predictive performance, we also used a built-in algorithm of feature selection in each model. Redundant features were removed using this algorithm. In addition, we compared the selected features with those from previous studies.

The association tests to identify the relevancy were conducted based on the data type. For categorical features, we used the Fisher exact test. For continuous features, the association test depended on the distributions in each class using the Kolmogorov-Smirnov normality test. Continuous features that were normally distributed in both classes (*P*≥.05) would be tested by an independent *t* test. If the variance was equal (*P*≥.05), we used the pooled method. Otherwise, we used the Satterthwaite method. For continuous features that were not normally distributed (*P*<.05), we used the Wilcoxon rank test. The features were significantly associated with the class if *P*<.05.

In addition to the association tests for scheme-independent feature selection or the filter method, we also conducted scheme-specific feature selection or the wrapper method using built-in algorithms in models as described in the Model Development section. Details on the algorithms of feature selection were meticulously described in Witten et al [32]. Complex model configurations, including to apply the algorithms, can be reproduced by entering the configuration code for each model (see Multimedia Appendix 1).

### Model Development

We used Weka (Waikato Environment for Knowledge Analysis) 3.8.3 (The University of Waikato, Hamilton, NZ) to develop machine learning models. We chose this software because of its practical ability to compare multiple models at once. The predictive performance of a machine learning model can be affected by its configuration uncertainty. Considering this issue, we used an add-on package of Weka—Auto-Weka 2.6.1 (The University of British Columbia, Vancouver, CA). It automatically selects the best machine learning model [33]. Its algorithm optimizes the configuration of each model within a predefined time period based on a predefined evaluation metric. We defined the time period as 12 hours and the metric as the AUC. However, this package shows only the best model, which is not necessarily a white-box model that is easier for humans to understand. Therefore, we also manually selected the best among 23 white-box models. These models were in a default configuration. Details on configurations for automatically and manually selected models were described (see Multimedia Appendix 1).

Manual selection to decide the best white-box model consisted of three steps. In step 1, we analyzed models that had greater or equal predictive performance compared to the logistic regression as the baseline. We used a corrected resampled *t* test, which was modified from the conventional paired *t* test, as previously developed [32]. The modification was intended to correct the significance of the difference in each evaluation metric that increases because of an increasing *k* fold. To calculate the *t* statistic (see Equation 1), we calculated the difference (∆*µ* = *µ*_j1_ – *µ*_j2_) between the means of the metric from the first model (*µ*_j1_) and those from the second model (*µ*_j2_) trained by *i*_k_ and validated by *j*_k_ from *k*-fold validation as described in the Model Validation section. The variance was estimated by the average of the squared differences between the *j*_k_ metric for each model and the mean of both models: *σ*_δ_^2^ = (∑ [*x*_j1_ − *µ*_j_] + ∑ [*x*_j2_ − *µ*_j_]) ÷ (2 × *n*_j_). The number of instances for the validation set was denoted as *n*_j_.

*t* = ∆*µ* ÷ √ [ ( 1 ÷ *k* + *n*_j_ ÷ *n*_i_ ) × *σ*_δ_^2^ ] (1)

In step 2, after the list of compared models no longer shrank using the *t* test, we used interval estimates with a decimal point precision to further shrink it. In the last step, we chose the best model by focusing on its sensitivity, interpretability, and trade-off between sensitivity and specificity.

Since customization is not provided by Weka in some circumstances, we optimized the best model from the manual selection by determining a custom threshold. All subjects of the dataset were used to determine an initial threshold. We then adjusted it by cross-validation to pursue expected sensitivity and specificity that were empirically reliable for unobserved data. Only training subsets were used to adjust the threshold, while validation subsets were only used to evaluate the predictive performances applying the predefined threshold. Details on the optimization procedure were also described (see Multimedia Appendix 1).

### Model Validation

Internal validation was conducted by repeated 10-fold cross-validation. The dataset was randomized and split up into 10 subsets with similar class balances. We used nine subsets to train a model in each fold, while the remaining subsets were used to validate it. We repeated these folds for 100 iterations with different seeds of randomization sequences. Cross-validation estimates the predictive performance of external validation [34]. This method of internal validation also improves the reliability of the reported predictive performance [35].

In addition, we also validated the best model with a custom threshold. The validation set consisted of 10 new subsets (n=35) taken from the original dataset (N=95) by stratified random sampling in SAS 9.4. The class balance was similar among subsets. These subsets were used to customize a threshold in pursuit of expected sensitivity and specificity that were reliable in most of the subsets.

### Evaluation Metrics

We applied multiple metrics to the model evaluation. These were calculated from a confusion matrix, which consists of true positives (TPs), true negatives (TNs), false negatives (FNs), and false positives (FPs). We calculated all of these metrics from recent studies because all of the metrics had not been reported. We inferred a confusion matrix from each study based on their sensitivity, specificity, and sample size of either positives (Ps) or negatives (Ns) (see Equations 2-5).

TP = P × Sensitivity (%) (2)

FN = P – TP (3)

TN = N × Specificity (%) (4)

FP = N – TN (5)

Point and interval estimates were used for comparison of each evaluation metric. Model selection was evaluated by the AUC, the area under the precision-recall curve (PRC), accuracy (see Equation 6), and sensitivity (see Equation 7). In addition, we evaluated the Akaike information criterion (AIC) to describe the trade-off between predictive performance and risk of overfitting relatively among models in the end of selection. The corrected AIC (AIC_C_) was used, considering the small training set, as previously described [36,37]. The best model was also evaluated by a calibration plot. We then demonstrated an ROC curve of the well-calibrated model. Comparing our model to those from recent studies, we used the AUC, sensitivity, and specificity (see Equation 8), in addition to the selected metric, which was the MCC (see Equation 9), because those metrics were widely used. However, an evaluation by the MCC prevents misleading predictive performances, particularly in a model developed from datasets with imbalanced classes [29]. Class imbalance is a common situation in preeclampsia and IUGR studies. In this situation, the MCC can provide a fair evaluation when comparing prediction models in order to choose the one that shows optimal performances on both sensitivity and specificity.

Accuracy (%) = ( TP + TN ) ÷ ( TP + FN + TN + FP ) × 100% (6)

Sensitivity (%) = ( TP ) ÷ ( TP + FN ) × 100% (7)

Specificity (%) = ( TN ) ÷ ( TN + FP ) × 100% (8)

MCC = ( TP × TN – FN × FP ) ÷ √ ( P × [ TP + FP ] × N × [ TN + FN ] ) (9)

## Results

### Selected Features

Several features were selected based on a preliminary statistical analysis (see Table 1). Selected maternal characteristics were maternal weight before pregnancy, BMI values (kg/m^2^), and BMI categories (<25 kg/m^2^ vs ≥25 kg/m^2^). Other features included three measures of the RI-UtA, three measures of the PI-UtA, the presence or absence of a bilateral notch, sFlt-1, PlGF, and the sFlt-1/PlGF ratio. The best model was automatically selected by a correlation-based feature selection of subset evaluation. It was combined with a backward greedy stepwise search algorithm.

**Figure 1:**
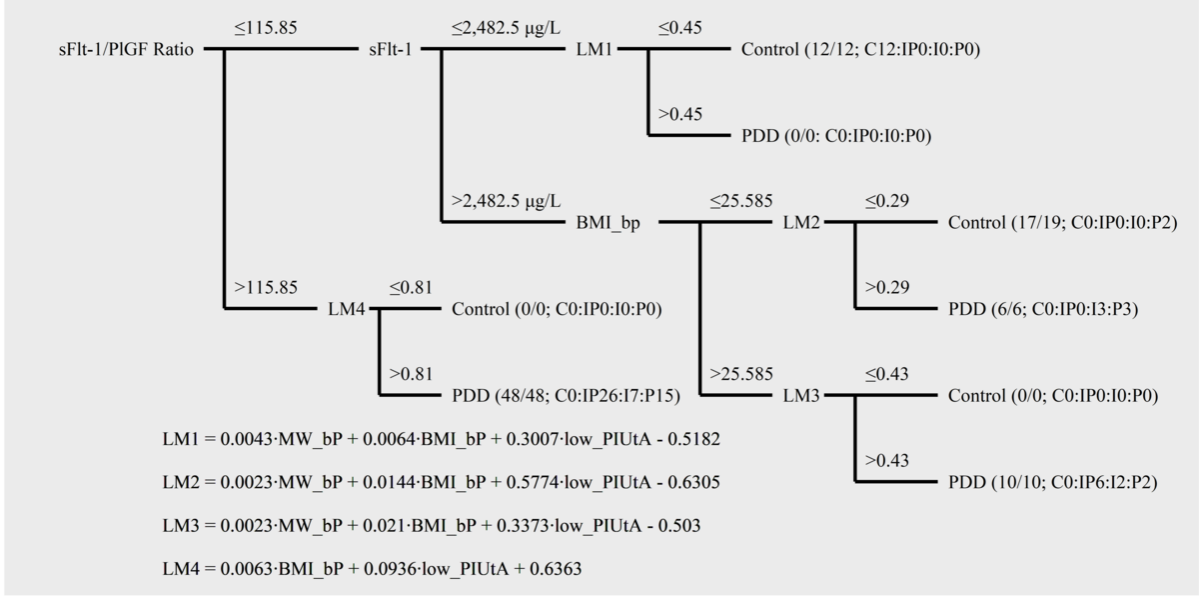
Characteristics of the classification via regression model using the lowest pulsatility index of the uterine artery (PI-UtA). Fractions in leaf nodes consist of true predicted numbers (numerators) and all predicted ones (denominators). A ratio of true predicted numbers is shown for control (C), both intrauterine growth restriction (IUGR) and preeclampsia (IP), IUGR only (I), and preeclampsia only (P). BMI_bP: body mass index before pregnancy (kg/m^2^); LM: linear model; low_PIUtA: the lowest pulsatility index of the uterine artery; MW_bP: maternal weight before pregnancy (kg); PDD: placental dysfunction–related disorder; PlGF: placental growth factor; sFlt: soluble fms-like tyrosine kinase receptor.

The selected features were extracted from mostly similar measures in recent studies (see Table 2). These were maternal characteristics, PI-UtA, sFlt-1, and PlGF, but not the bilateral notch. The sFlt-1/PlGF ratio turned out to be the most important feature in the best model (see Figure 1) as previously described [1,38,39].

However, the best model by manual selection was the right PI-UtA over the mean value. This choice is counterintuitive if the placental side is contralateral to the side on which the PI-UtA was measured. A previous study found that the PI-UtA was lower on the side ipsilateral to the placental side [40]. We then added the lowest value as a feature to provide an acceptable measure of the PI-UtA regardless of the placental laterality. We also demonstrated the proportion of the PI-UtA as the lowest value in either the right or left UtA (see Table 1). In this study, most of the lowest PI-UtA values were found in the right UtA (66/95, 69%).

**Table 1:** Descriptive and comparative analyses.

Feature			Class				*P* value	
			Control (n=29)		PDDs^a^ (n=66)			
**Maternal characteristics**								
	Maternal age (years), mean (95% CI)^b^			31.2 (30.9-31.5)		32.6 (32.4-32.7)		.23^c^
	**Parity, n (%)^d^**							**.10^e^**
		Nulliparous		15 (52)		47 (71)		
		Parous		14 (48)		19 (29)		
	Maternal weight (kg), median (IQR)^f^			58.0 (55.0-65.0)		68.0 (60.0-76.0)		.001^g,h^
	Maternal height (m), mean (95% CI)			1.66 (1.658-1.666)		1.65 (1.651-1.655)		.51^c^
	BMI (kg/m^2^), median (IQR)			21.6 (19.9-22.5)		24.4 (23.0-28.2)		<.001^g,h,i^
	**BMI, n (%)**							**.01^e,g^**
		<25 kg/m^2^		24 (83)		36 (55)		
		≥25 kg/m^2^		5 (17)		30 (45)		
**Uterine artery (UtA) Doppler measures, median (IQR)**								
	Right resistivity index (RI)-UtA			0.57 (0.49-0.61)		0.71 (0.63-0.78)		<.001^g,h^
	Left RI-UtA			0.59 (0.53-0.64)		0.73 (0.61-0.78)		<.001^g,h^
	Mean RI-UtA			0.57 (0.52-0.62)		0.71 (0.61-0.77)		<.001^g,h^
	Right pulsatility index (PI)-UtA			0.66 (0.60-0.71)		1.24 (0.79-1.56)		<.001^g,h,i^
	Left PI-UtA			0.70 (0.67-0.75)		1.33 (0.82-1.59)		<.001^g,h^
	Mean PI-UtA			0.68 (0.63-0.71)		1.26 (0.86-1.57)		<.001^g,h,i^
	Right peak systolic velocity (PSV)-UtA			58.30 (55.10-62.40)		59.25 (56.80-64.18)		.09^h^
	Left PSV-UtA			60.20 (59.10-64.10)		60.05 (57.10-63.80)		.99^h^
	Mean PSV-UtA			59.55 (58.25-61.40)		60.38 (57.54-64.06)		.31^h^
	**Bilateral notch, n (%)**							**<.001^e,g,i^**
		Nulliparous		0 (0)		47 (71)		
		Parous		29 (100)		19 (29)		
	Lowest PI-UtA, median (IQR)			0.65 (0.57-0.69)		1.16 (0.74-1.53)		<.001^g,h,j^
	**Laterality of lowest PI-UtA, n (%)**							**.23^e^**
		Right UtA		23 (79)		43 (65)		
		Left UtA		6 (21)		23 (35)		
**sFlt-1^k^ and PlGF ^l^, median (IQR)**								
	sFlt-1 (µg/L)			3014 (1852-4116)		13,961 (8893-22,218)		<.001^g,h,i^
	PlGF (µg/L)			626.9 (281.3-752.8)		68.4 (42.9-150.1)		<.001^g,h,i^
	sFlt-1/PlGF ratio			4.7 (2.6-15.1)		230.1 (100.8-483.0)		<.001^g,h,i^

^a^PDD: placental dysfunction–related disorder.

^b^Mean and 95% CI were calculated for numerical values with a normal distribution.

^c^Independent *t* test.

^d^Numbers and column proportions (%) were calculated for categorical values.

^e^Fisher exact test.

^f^Median and IQR were calculated for numerical values without a normal distribution.

^g^Statistically significant (alpha=.05).

^h^Wilcoxon rank test.

^i^Selected feature for the best model from automatic selection.

^j^Used for manual selection only.

^k^sFlt-1: soluble fms-like tyrosine kinase receptor-1.

^l^PlGF: placental growth factor.

**Table 2:** Features used by the models in this study compared to those from previous studies.^a^

Source			Gestational age at prediction		Features, n (for maternal characteristics) or + (used by the model) or – (not used by the model)												
					Maternal characteristics		MAP^b^		PI-UtA^c^		Bilateral notch		sFlt-1^d^		PlGF^e^		PAPP-A^f^
**This study**																	
	CVR^g^1 (right PI-UtA)	24-37 weeks		2		–		+		–		+		+		–	
	CVR2 (mean PI-UtA)	24-37 weeks		2		–		+		+		+		+		–	
	CVR3 (lowest PI-UtA)	24-37 weeks		2		–		+		–		+		+		–	
	158-tree random forest	24-37 weeks		1		–		+		+		+		+		–	
**Previous studies**																	
	Wright A et al (2019) [26]	11-13 weeks		10		–		+		–		+		+		–	
	Wright D et al (2019) [27]	11-13 weeks		11		+		+		–		+		+		–	
	Tan MY et al (2018) [25]	11-13 weeks		11		+		+		–		–		+		–	
	Sonek J et al (2018) [24]	11-13 weeks		10		–		+		–		–		+		+	
	Perales A et al (2017) [23]	27-28 weeks		3		+		–		–		+		+		–	
	Nuriyeva G et al (2017) [22]	11-13 weeks		N/A^h^		–		+		–		–		+		+	
	O'Gorman N et al (2017) [21]	11-13 weeks		11		+		+		–		–		+		+	
	Gallo DM et al (2016) [18]	19-24 weeks		11		+		+		–		+		+		–	
	Tsiakkas A et al (2016) [19]	30-34 weeks		11		–		–		–		+		+		–	
	Andrietti S et al (2016) [20]	35-37 weeks		11		+		+		–		+		+		–	
	O'Gorman N et al (2016) [17]	11-13 weeks		10		+		+		–		–		+		+	
	Wright D et al (2015) [16]	11-13 weeks		11		–		–		–		–		–		–	

^a^Models that showed the best sensitivity and an acceptable specificity in each study.

^b^MAP: mean arterial pressure.

^c^PI-UtA: pulsatility index of the uterine artery.

^d^sFlt-1: soluble fms-like tyrosine kinase receptor-1.

^e^PlGF: placental growth factor.

^f^PAPP-A: pregnancy-associated plasma protein-A.

^g^CVR: classification via regression.

^h^N/A: not applicable.

### Selected Machine Learning Models

We focused on the sensitivity to ensure minimum miss rates, which should improve maternal and neonatal outcomes. This resulted in the seven best machine learning models as shown in Table 3. The best model was the random forest from automatic selection; however, it is not a white-box model. We then also manually selected the best white-box model.

Classification via regression (CVR) classifies an outcome based on an M5P regression algorithm. It combines a pruned decision tree with smoothed linear models. There is also a built-in algorithm in CVR for selecting important features. A feature at the root node of the decision tree is the most important. Each leaf node has different linear models (LMs), which can be set to use different thresholds [32]. Optimization of this model was conducted by determining these thresholds (see Multimedia Appendix 1).

We developed CVR using only the mean values of UtA Doppler measures, in addition to this model using the right PI-UtA. We also developed CVR using the lowest PI-UtA value without other UtA Doppler measures. In the end, the model using the lowest PI-UtA value (see Figure 1) was the best, followed by that using either the right or mean PI-UtA (see Multimedia Appendices 2 and 3). We provided an interactive interface for readers to apply the model using the lowest PI-UtA value (see Multimedia Appendix 4).

We demonstrated characteristics of the best CVR using selected features from all subjects of the dataset (see Figure 1). LM1, LM3, and LM4 perfectly classified outcomes. However, a subpopulation of subjects was misclassified as the control instead of as having isolated preeclampsia. It consisted of subjects with sFlt-1/PlGF of ≤115.85, sFlt-1 of >2482.5 µg/L, and a BMI of ≤25.585 kg/m^2^.

**Figure 2:**
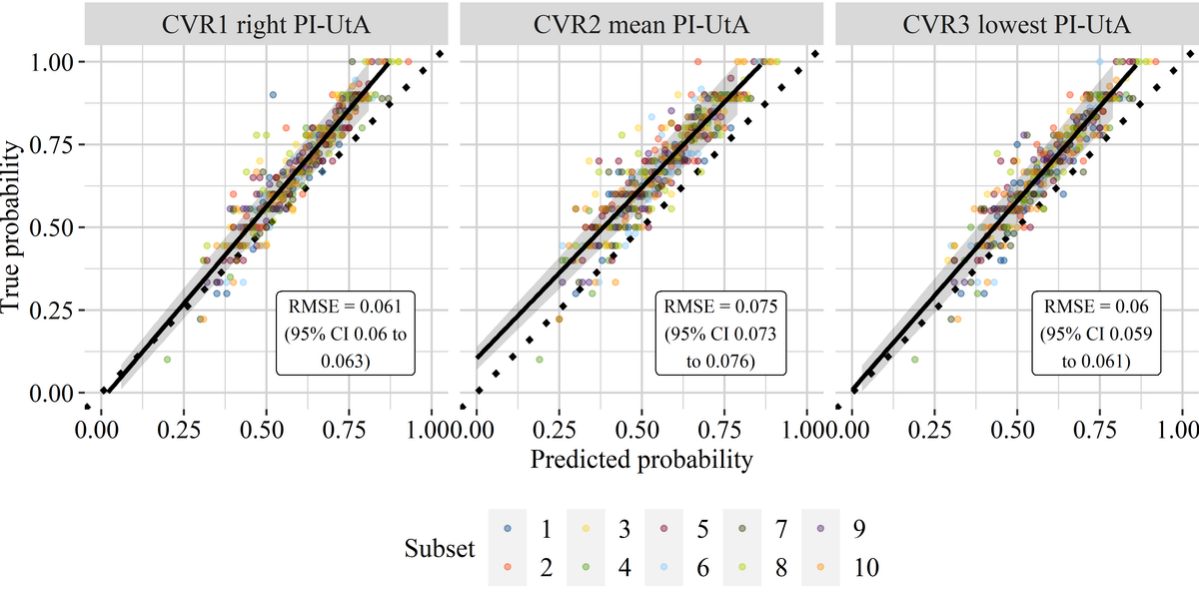
Calibration plots of classification via regression (CVR) models using the lowest, right, and mean pulsatility index of the uterine artery (PI-UtA). Each point demonstrates a validation subset taken from repeated 10-fold cross-validation. Colors denote subsets from stratified random sampling. RMSE: root mean square error.

Calibration plots are shown for CVR models using different types of PI-UtA (see Figure 2). Positive samples gathered higher values of both predicted and true probabilities from all of the CVR models. Then, classification biases were higher on positive samples from these models. However, all of the biases remained low because the root mean square error (RMSE) was only 0.076 at the maximum upper bound of the subsets, particularly from CVR using the mean PI-UtA. Therefore, these models were well calibrated. They also indicated robust positive predictive values (PPVs) or information retrieval (IR) precision.

**Figure 3:**
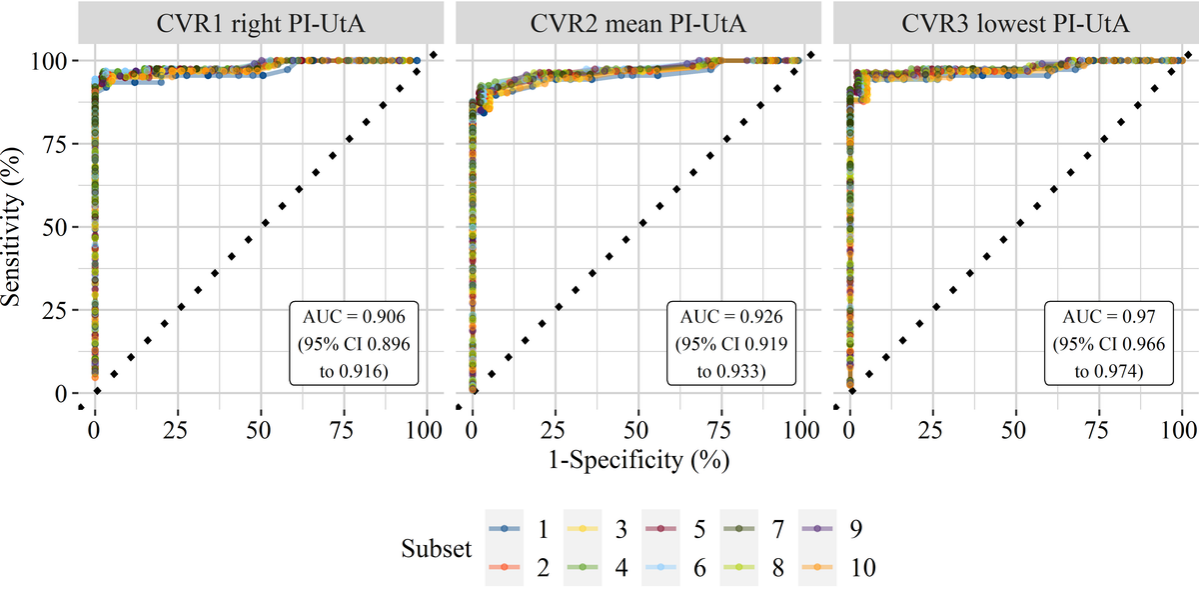
Receiver operating characteristic (ROC) curves of classification via regression (CVR) models using the lowest, right, and mean pulsatility index of the uterine artery (PI-UtA). Each ROC curve demonstrates a validation subset taken from repeated 10-fold cross-validation. Colors denote subsets from stratified random sampling. AUC: area under the receiver operating characteristic curve.

ROC curves are also shown for the CVR models (see Figure 3). C-statistics of 10 subsets are represented by an AUC that is shown for each CVR model. An average sensitivity was calculated for each distinct value of FP rates in order to measure the AUCs. The greatest AUC was for the CVR model that used the lowest PI-UtA (see Table 4). It significantly differs from that of the model using the right or mean PI-UtA value. Applying different thresholds for each LM, each CVR model has an acceptable trade-off between sensitivity and specificity without compromising its MCC.

**Table 3:** The seven best machine learning models.

Model		Performance metrics and rank				
		Area under the ROC^a^ curve	Area under the PRC^b^	Accuracy (%)	∆_i_ AIC_C_^c^	Sensitivity (%)
Automatic selection: random forest		0.976 (1)	0.958 (1)	92.6 (1)	0 (1)	90.7 (1)
**Manual selection**						
	CVR^d^	0.954 (5)	0.922 (3)	90.6 (4)	15 (4)	89.7 (2)
	Naïve Bayes	0.960 (2)	0.928 (2)	90.2 (5)	25 (5)	89.0 (3)
	Simple logistic	0.958 (3)	0.921 (4)	90.9 (2)	6 (2)	88.2 (4)
	Logistic model tree	0.957 (4)	0.920 (5)	90.8 (3)	7 (3)	88.0 (5)
	Multi-class classifier	0.932 (6)	0.868 (6)	89.9 (6)	30 (6)	86.8 (6)
	Logistic regression	0.932 (7)	0.868 (7)	89.9 (7)	30 (7)	86.8 (7)

^a^ROC: receiver operating characteristic.

^b^PRC: precision-recall curve.

^c^AIC_C_: corrected Akaike’s information criterion (∆_i_ AIC_C_ = AIC _Ci_ – AIC _C min_).

^d^CVR: classification via regression.

**Table 4:** Predictive performances shown by models in this study compared to those from recent studies.^a^

Source		Predictive performance^b^		
		AUC^c^	Sensitivity, %	Specificity, %
**This study**				
	CVR^d^1 (right PI-UtA^e^)	0.906 (0.896-0.916)	91 (85-96)	97 (90-100)
	CVR2 (mean PI-UtA)	0.926 (0.919-0.933)	95 (91-100)	100 (100-100)
	CVR3 (lowest PI-UtA)	0.970 (0.966-0.974)	95 (91-100)	100 (100-100)
	158-tree random forest	0.976 (0.967-0.985)	91 (87-94)	93 (92-95)
**Recent studies**				
	Wright A et al (2019) [26]	N/A^f,g^	85 (72-94)	90 (90-90)
	Wright D et al (2019) [27]	0.970 (0.950-0.990)	93 (76-99)	90^h^
	Tan MY et al (2018) [25]	N/A^g^	90 (80-96)	90^h^
	Sonek J et al (2018) [24]	N/A^g^	85^i^	95^i^
	Perales A et al (2017) [23]	0.930^i^	81^i^	95^i^
	Nuriyeva G et al (2017) [22]	0.888^i^	76^i^	90^i^
	O'Gorman N et al (2017) [21]	0.987^i^	100 (80-100)	90^h^
	Gallo DM et al (2016) [18]	0.930 (0.892-0.968)	85 (74-93)	90^h^
	Tsiakkas A et al (2016) [19]	0.987 (0.980-0.994)	100 (92-100)	90^h^
	Andrietti S et al (2016) [20]	0.938 (0.917-0.959)	82 (70-91)	90^h^
	O'Gorman N et al (2016) [17]	0.907^i^	89 (79-96)	90^h^
	Wright D et al (2015) [16]	0.811^i^	67 (59-74)	90^h^

^a^Models that showed the best sensitivity and an acceptable specificity in each study.

^b^Point and interval estimates.

^c^AUC: area under the receiver operating characteristic (ROC) curve.

^d^CVR: classification via regression.

^e^PI-UtA: pulsatility index of the uterine artery.

^f^N/A: not applicable because it was not available.

^g^This study showed an ROC curve without an AUC statement.

^h^Fixed specificity in order to define sensitivity.

^i^This study did not report an interval estimate.

### Comparison of Predictive Performances

**Figure 4:**
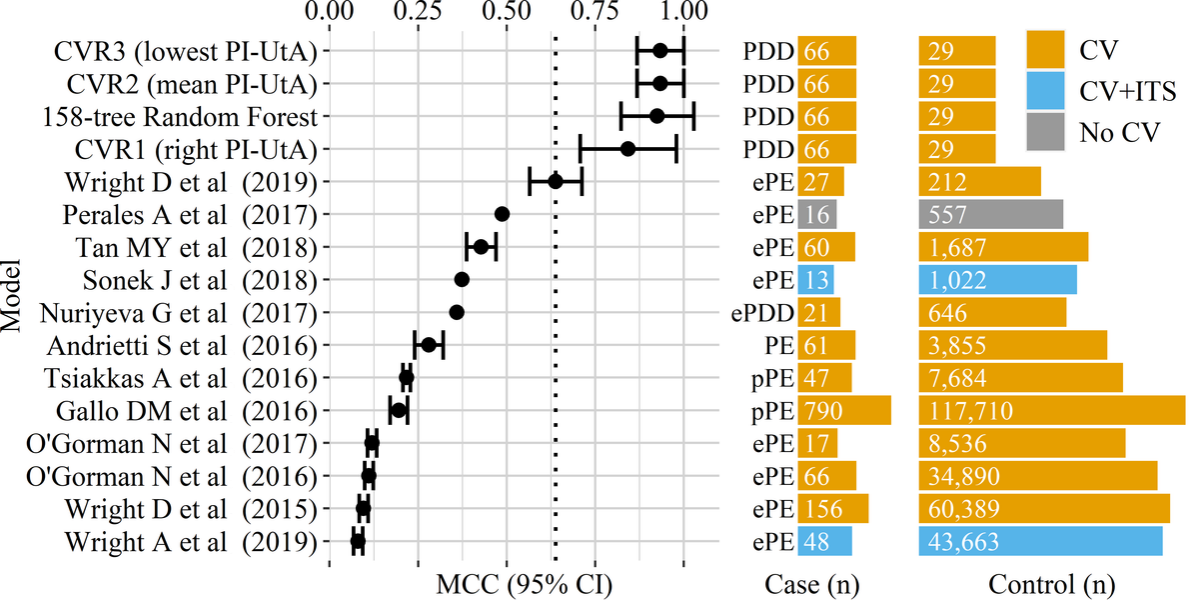
The Matthew correlation coefficient (MCC) and class balance. Control samples did not include other subtypes of either hypertension in pregnancy or placental dysfunction–related disorders (PDDs). Colors denote validation methods. Several studies did not report interval estimates and/or cross-validation (CV). To improve visualization, the scales for either case or control sample sizes were individually log-transformed. CVR: classification via regression; ePDD: early placental dysfunction–related disorder; ePE: early preeclampsia; ITS: independent test set; PE: preeclampsia; PI-UtA: pulsatility index of the uterine artery; pPE: preterm preeclampsia.

The CVR model with the lowest PI-UtA value was found to achieve the most robust predictive performance (see Figure 4 and Table 4), as determined by the MCC (.93, 95% CI .87-1.00). The MCC of this model showed no difference compared to that of either the best model from automatic selection (.93, 95% CI .82-1.00) or the CVR model with the mean PI-UtA value (.93, 95% CI .87-1.00). However, the MCC of this CVR model was higher than those from the models with the right PI-UtA value (.84, 95% CI .71-.98). The predictive performance in this study was assessed by cross-validation without an independent test set, similar to most of the recent studies. However, we developed our models from a dataset with a class balance that was better than those of recent studies. The MCCs of our models were also higher than those of recent studies (see Figure 4 and Multimedia Appendix 3). Compared to random forest with the best AIC (see Table 3), the CVR models with the lowest, right, and mean PI-UtA showed AIC values of 13, 15, and 17, respectively.

Comparison of predictive performances was also described using other evaluation metrics that are commonly used (see Table 4). There was significant difference in the AUC between the CVR models that used the lowest and other PI-UtA values. Meanwhile, the CVR model with the lowest PI-UtA value was not significantly different compared to the automatically selected 158-tree random forest. From recent studies, Wright et al [27] and Tsiakkas et al [19] showed models with more competitive areas under the ROC than those of our models. However, our models show sensitivities and specificities that are not inferior compared to those from recent studies. In addition, our models were developed by a dataset with a better class balance, whose case class size was 69% (66/95), compared to the most balanced dataset from Wright et al [27], whose case size was 11.3% (27/239) (see Figure 4).

## Discussion

### Principal Findings

The best model in this study was a CVR one that used the lowest PI-UtA values. It was an acceptable model, because the lowest PI-UtA value was reliably found ipsilateral to the placental side [40]. This model demonstrated higher MCCs and PPVs, but not sensitivity or AUC, compared to those from previous studies (see Figure 4, Table 4, and Multimedia Appendix 3). MCC was intended for achieving our goal to eventually avoid mortality and morbidity and unnecessary health care costs. This may result in improved maternal and neonatal outcomes. It also outperformed models from recent studies in terms of specificity. Compared to a model that had 90% specificity, this potentially reduces 10% of health care costs. Applying a predictive model that uses the sFlt-1/PlGF ratio, a previous study showed a similar reduction in health care costs [41]. Even without considering the health economics, the MCC is still practical to consider FPs along with other components of the confusion matrix, which reflect numbers of false referral decisions on predicted preeclampsia and IUGR. Making wrong decisions may harm pregnant women, especially in developing countries where a distant and dangerous journey must be taken by pregnant women to reach higher-level health care facilities. Therefore, a CVR model that used the lowest PI-UtA values was better in compromising between the mortality and morbidity and costs compared to the those of other models in either this study or previous studies.

### Comparison With Prior Work

The selected features were consistent with those from previous studies. The preeclampsia risk was found to be higher in women with a prepregnancy BMI classified as overweight or obese compared to those classified as underweight or normal (with a cutoff of ≥24 kg/m^2^) [42]. This disease was also associated with combinations of a bilateral notch, both RI-UtA and PI-UtA, and sFlt-1/PlGF measures in the second or third trimester [43,44]. However, these combinations were inconsistently associated with the IUGR with or without preeclampsia [45-47]. As to the UtA Doppler measures, no association was found between placental location and either preeclampsia or a low birth weight [48]. Using features corresponding to results from previous studies, an acceptable machine learning model can be developed.

CVR belongs to a group of superior meta-classifiers for predicting malicious cyberattacks, but it was not the best as a bagging classifier [49]. In this study, the bagging classifier did not outperform CVR. The optimized CVR model was also better than the random forest from automatic selection. Surprisingly, this model was not outperformed by any state-of-the-art machine learning models. Those included both artificial neural networks and support vector machines. These models were also candidates for automatic selection in this study. One possible reason is because of a regression model used by CVR that divides the dataset into several subpopulations using a decision tree. In the field of medicine, this algorithm is widely known as a reliable and effective machine learning application [50].

Each leaf node in the decision tree has a different LM. It can capture different correlations among features in each subpopulation that is normally distributed [51]. Different thresholds for each LM may approach heterogeneity in PDDs, especially in preeclampsia. Thresholds or cutoffs also give more understanding as to how outcomes are predicted. Thus, this model has the interpretability that we intended to achieve.

In this study, the CVR models split subjects by an sFlt-1/PlGF ratio of 115.85. This cutoff was higher than 38 as previously described [38,39]. This is reasonable, because predicted outcomes in this study were not only preeclampsia but also IUGR. Birth weights showed no difference for babies from women with IUGR that were classified by 38 as a cutoff for the sFlt-1/PlGF ratio [47]. Therefore, a different cutoff for the sFlt-1/PlGF ratio is related to predicted outcomes in this study that differed from those of previous studies.

PIs were also selected by the CVR models of UtA Doppler measures. Unexpectedly, one of the CVR models in this study chose the right PI-UtA instead of the mean value, which is conventionally used [27,44,47]. This is counterintuitive because of placental laterality, although a previous study showed no difference between the right and left PI-UtA values (*P*=.20) [52]. However, the CVR model using the lowest value had a higher MCC than that using the right PI-UtA in this study. A previous model demonstrated a greater AUC when using the lowest PI-UtA instead of the mean or highest value [53]. This is also more acceptable, because the lowest PI-UtA value was shown to be ipsilateral to the placental location [40]. Thus, this measure is independent of placental laterality.

However, between the CVR model using the right PI-UtA and the one using the lowest value, we may also consider several similarities. These were shown by most of the evaluation metrics and characteristics. The similarities may be coincidental because most of the subjects had the lowest value on the right side of the UtA in this study (66/95, 69%; see Table 1). Most placentas were located on the right side (57.4%) compared to the middle (22.2%) and left side (20.4%) on the anterior uterine wall [54]. Interestingly, the sleeping position before becoming pregnant was mostly right lateral by pregnant women with a placenta on either the anterior, lateral, or fundal uterine wall (*P*=.001) [55].

In addition to the lowest and the right PIs, the CVR model using the mean PI-UtA value also demonstrated a competitive predictive performance. This model showed each LM using a combination of the mean PI-UtA and bilateral notch. Apparently, both of them are a counterpart of the lowest or the right PI-UtA alone in each LM of other CVR models. The predictive value of the mean PI-UtA was found to be higher if the bilateral notch was present compared to when it was absent [43]. Nevertheless, this model demonstrated the highest RMSE compared to CVR models using the lowest or the right PI-UtA (see Figure 2). Therefore, the best model in this study was the CVR model that used the lowest PI-UtA.

The best model used 25.585 kg/m^2^ as a cutoff for BMI in its decision tree. This is similar to the cutoff for BMI as a risk factor of preeclampsia [42]. As indicated by each LM in the best model, an effect on PDDs was partially contributed by the two maternal characteristics of maternal weight and the BMI. However, the risk of preeclampsia, as a subtype of PDD, was adjusted by multiple factors instead of only these anthropometrics [56]. Other maternal characteristics were not represented in the dataset we used. So, our models need further improvement using a dataset with more maternal characteristics.

None of the predictive models from 12 recent studies outperformed our models according to the MCC [16-27]. All of those studies used datasets with highly imbalanced classes that may have masked the misclassification of positive samples [29]. There are many aspects that may cause similar problems [3,4,28]. These include an outcome leakage that was encountered by some of those studies [18,20,23]. Mean arterial pressure (MAP) may easily infer the class because it is calculated from the same measures as for the diagnostic criteria of preeclampsia. This is true if MAP is taken in the second trimester, when it is used for predicting either early or preterm preeclampsia. This feature may also cause an outcome leakage if it is taken at 35-37 weeks’ gestation, when it is used for predicting late preeclampsia. Outcome leakage causes the predictive performance to be overoptimistic [30].

### Strengths

To the best our knowledge, this is the first study that used machine learning to predict preeclampsia and/or IUGR using features in the second or third trimester of pregnancy. Our models outperformed 12 recent studies according to the MCC. This study also used a dataset with a better class balance than those used by recent studies as well as the size of the case class. Predicting preeclampsia [26,27] and IUGR [47] used to be developed using conventional statistical modeling. A previous study developed a machine learning model (ie, multilayer perceptron) for predicting PDDs in the first trimester [22]. However, its PPV or IR precision was insufficient. Other studies developed a machine learning model to characterize gene expression of preeclampsia as mechanism studies instead of for prediction [4,57]. Yet, a machine learning model can both perform a robust prediction and reveal mechanisms of a disease.

### Limitations

A pitfall should be considered when applying our models. They do not distinguish between preeclampsia and IUGR. These models should only be applied for a referral decision. This means whether a clinician should refer the pregnant women to a hospital with advanced maternal and neonatal care within a certain time period [8]. For pregnant women who will develop preeclampsia with or without IUGR before term, advanced maternal care will be needed for cesarean section. It is one of the possible modes for early delivery that was recommended at any time in deteriorated maternal or fetal condition [13]. Meanwhile, for pregnant women who will develop IUGR with or without preeclampsia, the advanced neonatal care will be needed for the babies. They were found having low birth weight and more in-hospital deaths, even among those who were delivered at term [14,15].

Other applications of our models exclude a decision of delivery before term. This decision should be made based on models that specifically predict severe cases of early-onset or preterm preeclampsia and IUGR. It is because a false decision on early delivery will bring unnecessary prematurity. Nonetheless, no prediction for isolated preeclampsia is needed for those at term since no prematurity will occur as a consequence of early delivery decision.

Controls in this study also did not include other subtypes of hypertension in pregnancy. They may be indistinguishable from PDDs, but there is no need for patient referral. There is a possibility that more FPs will occur in subjects who will develop other subtypes of this disease. Therefore, the clinical impact may be unnecessary patient referral to higher-level health care facilities.

We also need to conduct external validation to confirm predictive performance of our models. There is a possibility that these models overfit the dataset. This is still possible even though they were evaluated by sufficient cross-validation because of consideration of diverse phenotypes of preeclampsia, other subtypes of hypertension in pregnancy, and other PDDs.

### Conclusions

CVR is a machine learning model that has robust predictive performance in classifying PDDs versus a control group. This model differentiates PDDs from a control that has no other subtypes of hypertension in pregnancy. Using features in the second or third trimester, this model may be reliable for countries with low numbers of first visits in the first trimester, but further investigations are needed. Although the best preventive method for preeclampsia is not in the second or third trimester, this model can still be beneficial in the context of clinical management.

## Appendix

Multimedia Appendix 1 Automatic and manual model selection.

Multimedia Appendix 2 Characteristics of classification via regression (CVR) models with the right and mean of the pulsatility index of the uterine artery (PI-UtA).

Multimedia Appendix 3 Evaluation metrics and validation method for comparison with recent studies.

Multimedia Appendix 4 Interactive model.

## Abbreviations

AIC: Akaike information criterion
AIC_C_: corrected Akaike information criterion
AUC: area under the receiver operating characteristic curve
CVR: classification via regression
FN: false negative
FP: false positive
IR: information retrieval
IUGR: intrauterine growth restriction
LM: linear model
MAP: mean arterial pressure
MCC: Matthew correlation coefficient
MOE: Ministry of Education
MOST: Ministry of Science and Technology
N: negative
P: positive
PDD: placental dysfunction–related disorder
PI: pulsatility index
PI-UtA: pulsatility index of the uterine artery
PlGF: placental growth factor
PPV: positive predictive value
PRC: precision-recall curve
PSV: peak systolic velocity
PSV-UtA: peak systolic velocity of the uterine artery
RI: resistivity index
RI-UtA: resistivity index of the uterine artery
RMSE: root mean square error
ROC: receiver operating characteristic
sFlt-1: soluble fms-like tyrosine kinase receptor-1
STROBE: STrengthening the Reporting of OBservational studies in Epidemiology
TN: true negative
TP: true positive
UtA: uterine artery
Weka: Waikato Environment for Knowledge Analysis

## References

[1] Kwiatkowski S, Dołegowska B, Kwiatkowska E, Rzepka R, Marczuk N, Loj B, Torbè A (2017). Maternal endothelial damage as a disorder shared by early preeclampsia, late preeclampsia and intrauterine growth restriction. J Perinat Med.

[2] Reijnders IF, Mulders AGMGJ, Koster MPH (2018). Placental development and function in women with a history of placenta-related complications: A systematic review. Acta Obstet Gynecol Scand.

[3] Fabjan-Vodusek V, Kumer K, Osredkar J, Verdenik I, Gersak K, Premru-Srsen T (2019). Correlation between uterine artery Doppler and the sFlt-1/PlGF ratio in different phenotypes of placental dysfunction. Hypertens Pregnancy.

[4] Nair TM (2018). Statistical and artificial neural network-based analysis to understand complexity and heterogeneity in preeclampsia. Comput Biol Chem.

[5] Abalos E, Cuesta C, Grosso AL, Chou D, Say L (2013). Global and regional estimates of preeclampsia and eclampsia: A systematic review. Eur J Obstet Gynecol Reprod Biol.

[6] Class QA, Rickert ME, Lichtenstein P, D'Onofrio BM (2014). Birth weight, physical morbidity, and mortality: A population-based sibling-comparison study. Am J Epidemiol.

[7] Nardozza LMM, Caetano ACR, Zamarian ACP, Mazzola JB, Silva CP, Marçal VMG, Lobo TF, Peixoto AB, Araujo Júnior E (2017). Fetal growth restriction: Current knowledge. Arch Gynecol Obstet.

[8] von Dadelszen P, Payne B, Li J, Ansermino JM, Broughton Pipkin F, Côté AM, Douglas MJ, Gruslin A, Hutcheon JA, Joseph KS, Kyle PM, Lee T, Loughna P, Menzies JM, Merialdi M, Millman AL, Moore MP, Moutquin J, Ouellet AB, Smith GN, Walker JJ, Walley KR, Walters BN, Widmer M, Lee SK, Russell JA, Magee LA, PIERS Study Group (2011). Prediction of adverse maternal outcomes in pre-eclampsia: Development and validation of the fullPIERS model. Lancet.

[9] Moller A, Petzold M, Chou D, Say L (2017). Early antenatal care visit: A systematic analysis of regional and global levels and trends of coverage from 1990 to 2013. Lancet Glob Health.

[10] Park F, Russo K, Williams P, Pelosi M, Puddephatt R, Walter M, Leung C, Saaid R, Rawashdeh H, Ogle R, Hyett J (2015). Prediction and prevention of early-onset pre-eclampsia: Impact of aspirin after first-trimester screening. Ultrasound Obstet Gynecol.

[11] Roberge S, Nicolaides K, Demers S, Hyett J, Chaillet N, Bujold E (2017). The role of aspirin dose on the prevention of preeclampsia and fetal growth restriction: Systematic review and meta-analysis. Am J Obstet Gynecol.

[12] Caillon H, Tardif C, Dumontet E, Winer N, Masson D (2018). Evaluation of sFlt-1/PlGF ratio for predicting and improving clinical management of pre-eclampsia: Experience in a specialized perinatal care center. Ann Lab Med.

[13] American College of Obstetricians and Gynecologists (2019). ACOG Practice Bulletin No. 202: Gestational hypertension and preeclampsia. Obstet Gynecol.

[14] Eskes M, Waelput A, Scherjon S, Bergman K, Abu-Hanna A, Ravelli A (2017). Small for gestational age and perinatal mortality at term: An audit in a Dutch national cohort study. Eur J Obstet Gynecol Reprod Biol.

[15] Ewing AC, Ellington SR, Shapiro-Mendoza CK, Barfield WD, Kourtis AP (2017). Full-term small-for-gestational-age newborns in the US: Characteristics, trends, and morbidity. Matern Child Health J.

[16] Wright D, Syngelaki A, Akolekar R, Poon LC, Nicolaides KH (2015). Competing risks model in screening for preeclampsia by maternal characteristics and medical history. Am J Obstet Gynecol.

[17] O'Gorman N, Wright D, Syngelaki A, Akolekar R, Wright A, Poon LC, Nicolaides KH (2016). Competing risks model in screening for preeclampsia by maternal factors and biomarkers at 11-13 weeks gestation. Am J Obstet Gynecol.

[18] Gallo DM, Wright D, Casanova C, Campanero M, Nicolaides KH (2016). Competing risks model in screening for preeclampsia by maternal factors and biomarkers at 19-24 weeks' gestation. Am J Obstet Gynecol.

[19] Tsiakkas A, Saiid Y, Wright A, Wright D, Nicolaides KH (2016). Competing risks model in screening for preeclampsia by maternal factors and biomarkers at 30-34 weeks' gestation. Am J Obstet Gynecol.

[20] Andrietti S, Silva M, Wright A, Wright D, Nicolaides KH (2016). Competing-risks model in screening for pre-eclampsia by maternal factors and biomarkers at 35-37 weeks' gestation. Ultrasound Obstet Gynecol.

[21] O'Gorman N, Wright D, Poon LC, Rolnik DL, Syngelaki A, Wright A, Akolekar R, Cicero S, Janga D, Jani J, Molina FS, de Paco Matallana C, Papantoniou N, Persico N, Plasencia W, Singh M, Nicolaides KH (2017). Accuracy of competing-risks model in screening for pre-eclampsia by maternal factors and biomarkers at 11-13 weeks' gestation. Ultrasound Obstet Gynecol.

[22] Nuriyeva G, Kose S, Tuna G, Kant M, Akis M, Altunyurt S, Islekel GH, Dogan OE (2017). A prospective study on first trimester prediction of ischemic placental diseases. Prenat Diagn.

[23] Perales A, Delgado JL, de la Calle M, García-Hernández JA, Escudero AI, Campillos JM, Sarabia MD, Laíz B, Duque M, Navarro M, Calmarza P, Hund M, Álvarez FV, STEPS investigators (2017). sFlt-1/PlGF for prediction of early-onset pre-eclampsia: STEPS (Study of Early Pre-eclampsia in Spain). Ultrasound Obstet Gynecol.

[24] Sonek J, Krantz D, Carmichael J, Downing C, Jessup K, Haidar Z, Ho S, Hallahan T, Kliman HJ, McKenna D (2018). First-trimester screening for early and late preeclampsia using maternal characteristics, biomarkers, and estimated placental volume. Am J Obstet Gynecol.

[25] Tan MY, Wright D, Syngelaki A, Akolekar R, Cicero S, Janga D, Singh M, Greco E, Wright A, Maclagan K, Poon LC, Nicolaides KH (2018). Comparison of diagnostic accuracy of early screening for pre-eclampsia by NICE guidelines and a method combining maternal factors and biomarkers: Results of SPREE. Ultrasound Obstet Gynecol.

[26] Wright A, Wright D, Syngelaki A, Georgantis A, Nicolaides KH (2019). Two-stage screening for preterm preeclampsia at 11-13 weeks' gestation. Am J Obstet Gynecol.

[27] Wright D, Tan MY, O'Gorman N, Poon LC, Syngelaki A, Wright A, Nicolaides KH (2019). Predictive performance of the competing risk model in screening for preeclampsia. Am J Obstet Gynecol.

[28] Agarwal V, Podchiyska T, Banda JM, Goel V, Leung TI, Minty EP, Sweeney TE, Gyang E, Shah NH (2016). Learning statistical models of phenotypes using noisy labeled training data. J Am Med Inform Assoc.

[29] Chicco D (2017). Ten quick tips for machine learning in computational biology. BioData Min.

[30] Luo W, Phung D, Tran T, Gupta S, Rana S, Karmakar C, Shilton A, Yearwood J, Dimitrova N, Ho TB, Venkatesh S, Berk M (2016). Guidelines for developing and reporting machine learning predictive models in biomedical research: A multidisciplinary view. J Med Internet Res.

[31] Premru-Srsen T (2018). Mendeley Data, v1.

[32] Witten IH, Frank E, Hall MA, Pal CJ (2017). Data Mining: Practical Machine Learning Tools and Techniques. 4th edition.

[33] Kotthoff L, Thornton C, Hoos HH, Hutter F, Leyton-Brown K (2017). Auto-WEKA 2.0: Automatic model selection and hyperparameter optimization in WEKA. J Mach Learn Res.

[34] Jung Y, Hu J (2015). A K-fold averaging cross-validation procedure. J Nonparametr Stat.

[35] Ounpraseuth S, Lensing SY, Spencer HJ, Kodell RL (2012). Estimating misclassification error: A closer look at cross-validation based methods. BMC Res Notes.

[36] Wagenmakers E, Farrell S (2004). AIC model selection using Akaike weights. Psychon Bull Rev.

[37] Brewer M, Butler A, Cooksley S (2016). The relative performance of AIC, AICC and BIC in the presence of unobserved heterogeneity. Methods Ecol Evol.

[38] Zeisler H, Llurba E, Chantraine F, Vatish M, Staff AC, Sennström M, Olovsson M, Brennecke SP, Stepan H, Allegranza D, Dilba P, Schoedl M, Hund M, Verlohren S (2016). Predictive value of the sFlt-1:PlGF ratio in women with suspected preeclampsia. N Engl J Med.

[39] Sabrià E, Lequerica-Fernández P, Ganuza PL, Ángeles EE, Escudero AI, Martínez-Morillo E, Alvárez FV (2018). Use of the sFlt-1/PlGF ratio to rule out preeclampsia requiring delivery in women with suspected disease. Is the evidence reproducible?. Clin Chem Lab Med.

[40] Chen Q, Izumi A, Minakami H, Sato I (1998). Comparative changes in uterine artery blood flow waveforms in singleton and twin pregnancies. Gynecol Obstet Invest.

[41] Frusca T, Gervasi M, Paolini D, Dionisi M, Ferre F, Cetin I (2017). Budget impact analysis of sFlt-1/PlGF ratio as prediction test in Italian women with suspected preeclampsia. J Matern Fetal Neonatal Med.

[42] Shao Y, Qiu J, Huang H, Mao B, Dai W, He X, Cui H, Lin X, Lv L, Wang D, Tang Z, Xu S, Zhao N, Zhou M, Xu X, Qiu W, Liu Q, Zhang Y (2017). Pre-pregnancy BMI, gestational weight gain and risk of preeclampsia: A birth cohort study in Lanzhou, China. BMC Pregnancy Childbirth.

[43] Afrakhteh M, Moeini A, Taheri MS, Haghighatkhah HR, Fakhri M, Masoom N (2014). Uterine Doppler velocimetry of the uterine arteries in the second and third trimesters for the prediction of gestational outcome. Rev Bras Ginecol Obstet.

[44] Tarasevičienė V, Grybauskienė R, Mačiulevičienė R (2016). sFlt-1, PlGF, sFlt-1/PlGF ratio and uterine artery Doppler for preeclampsia diagnostics. Medicina (Kaunas).

[45] Rizos D, Eleftheriades M, Karampas G, Rizou M, Haliassos A, Hassiakos D, Vitoratos N (2013). Placental growth factor and soluble fms-like tyrosine kinase-1 are useful markers for the prediction of preeclampsia but not for small for gestational age neonates: A longitudinal study. Eur J Obstet Gynecol Reprod Biol.

[46] Albu AR, Anca AF, Horhoianu VV, Horhoianu IA (2014). Predictive factors for intrauterine growth restriction. J Med Life.

[47] Kwiatkowski S, Bednarek-Jędrzejek M, Ksel J, Tousty P, Kwiatkowska E, Cymbaluk A, Rzepka R, Chudecka-Głaz A, Dołęgowska B, Torbè A (2018). sFlt-1/PlGF and Doppler ultrasound parameters in SGA pregnancies with confirmed neonatal birth weight below 10th percentile. Pregnancy Hypertens.

[48] Contro E, Maroni E, Cera E, Youssef A, Bellussi F, Pilu G, Rizzo N, Pelusi G, Ghi T (2010). Unilaterally increased uterine artery resistance, placental location and pregnancy outcome. Eur J Obstet Gynecol Reprod Biol.

[49] Michael G, Kumaravel A, Chandrasekar A (2015). Detection of malicious attacks by meta classification algorithms. Int J Adv Netw Appl.

[50] Podgorelec V, Kokol P, Stiglic B, Rozman I (2002). Decision trees: An overview and their use in medicine. J Med Syst.

[51] Lin L, Wang Q, Sadek AW (2016). A combined M5P tree and hazard-based duration model for predicting urban freeway traffic accident durations. Accid Anal Prev.

[52] Ergin RN, Yayla M (2015). Uterine artery pulsatility index and diastolic notch laterality according to the placental location. Clin Exp Obstet Gynecol.

[53] Poon LC, Staboulidou I, Maiz N, Plasencia W, Nicolaides KH (2009). Hypertensive disorders in pregnancy: Screening by uterine artery Doppler at 11-13 weeks. Ultrasound Obstet Gynecol.

[54] Hoogland HJ, de Haan J (1980). Ultrasonographic placental localization with respect to fetal position in utero. Eur J Obstet Gynecol Reprod Biol.

[55] Koken GN, Kanat-Pektas M, Kayman Köse S, Arioz DT, Yilmazer M (2014). Maternal blood pressure and dominant sleeping position may affect placental localization. J Matern Fetal Neonatal Med.

[56] Bartsch E, Medcalf KE, Park AL, Ray JG, High Risk of Pre-eclampsia Identification Group (2016). Clinical risk factors for pre-eclampsia determined in early pregnancy: Systematic review and meta-analysis of large cohort studies. BMJ.

[57] Zhang J, Simonti CN, Capra JA (2018). Genome-wide maps of distal gene regulatory enhancers active in the human placenta. PLoS One.

