# Supplementary material for: Prediction of Preeclampsia and Intrauterine Growth Restriction: Development of Machine Learning Models on a Prospective Cohort
Source: JMIR Med Inform. 2020 May 18;8(5):e15411. doi: 10.2196/15411 (PMC7265111; doi:10.2196/15411)
Supplement: Multimedia Appendix 1 [file medinform_v8i5e15411_app1.docx]

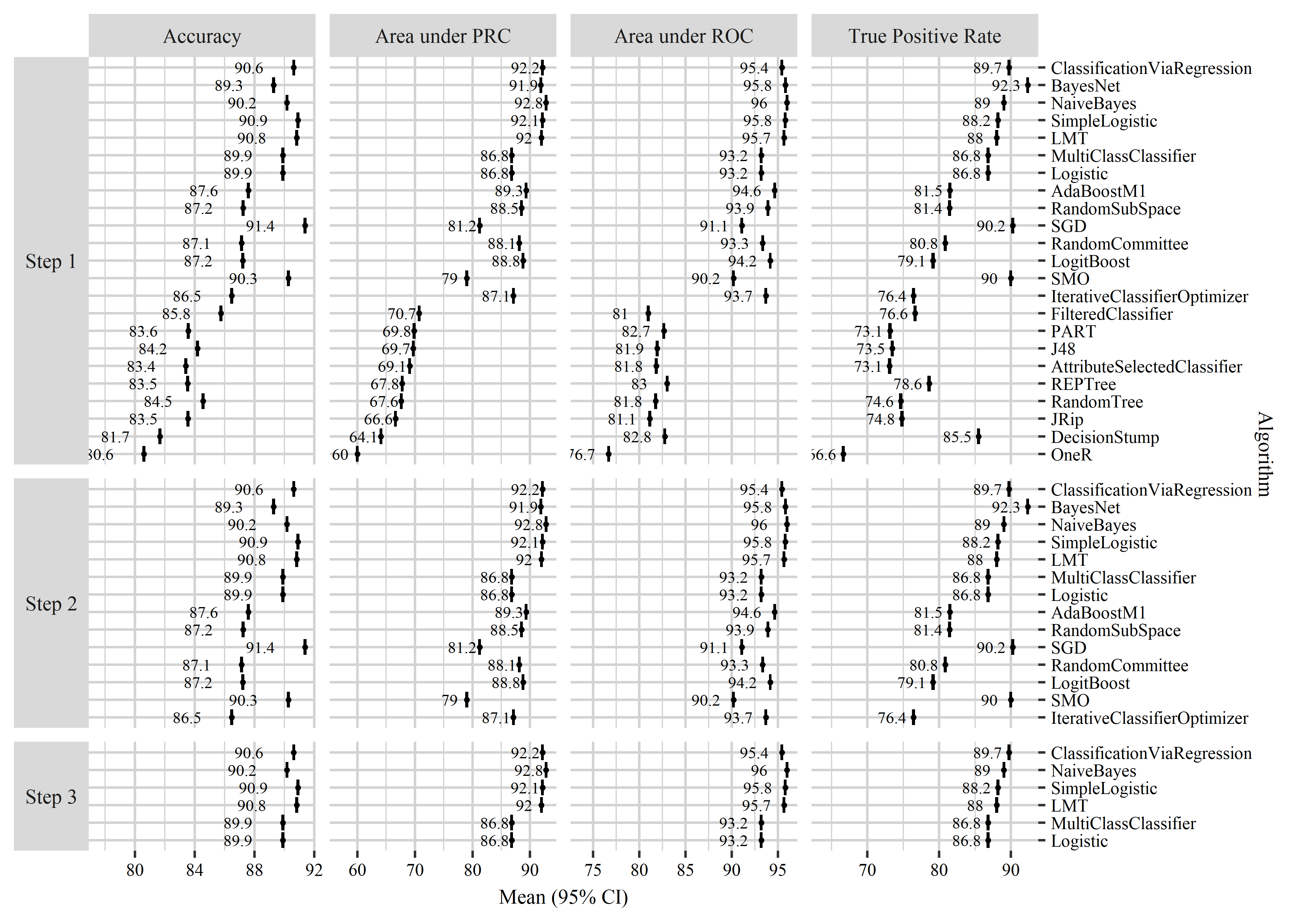
MULTIMEDIA APPENDIX 1

Automatic and manual model selection

# Prediction of preeclampsia and intrauterine growth restriction: development of machine learning models on a prospective cohort

Herdiantri Sufriyana^1,2^, MD, MSc; Yu-Wei Wu^1,3^, PhD; Emily Chia-Yu Su^1,3,4^, PhD

^1^Graduate Institute of Biomedical Informatics, College of Medical Science and Technology, Taipei Medical University, Taipei, Taiwan.

^2^Department of Medical Physiology, College of Medicine, University of Nahdlatul Ulama Surabaya, Surabaya, Indonesia.

^3^Clinical Big Data Research Center, Taipei Medical University Hospital, Taipei, Taiwan.

^4^Research Center for Artificial Intelligence in Medicine, Taipei Medical University, Taipei, Taiwan.

Figure S1. Comparison of 23 white-box models developed by machine learning. Each step is described in the main text. The models are ordered by minimum value among evaluation metrics in each model.

We used WEKA 3.8.3 (The University of Waikato, Hamilton, NZ) to develop prediction model. The best machine learning model was automatically selected by Auto-WEKA 2.6.1 (University of British Columbia, Vancouver, CA) in addition to manual selection of 23 white-box models with default configuration.

In automatic model selection, the algorithm optimized the configuration of each model. This was based on area under the receiver operating characteristics curve. By initial random model and its configuration, this algorithm was set to stop after 12 h.

In manual model selection, predictive performance of white-box models was compared by accuracy, area under receiver operating characteristics (ROC) curve, area under precision-recall curve (PRC), and sensitivity (Figure S1). Minimum value was chosen among these evaluation metrics in each model as the value for sorting them into descending order.

We show comparison in each step as described in the main text to easily denote how the list of models is shrinking. This comparison also shows which models having higher value in each metric compared with logistic regression in each step. In the end, we have got six white-box models as shown concisely in the main text beyond the best one from automatic selection.

Table S1. Configuration code for each model.

| Modelling | Configuration code |
| --- | --- |
| **Automatic model selection ^a^** |  |
| A - Optimization algorithm | weka.classifiers.meta.AutoWEKAClassifier -seed 123 -timeLimit 720 -memLimit 4096 -nBestConfigs 10 -metric errorRate -parallelRuns 2 |
| B - Selected filter method |  |
| a - Attribute evaluator | weka.attributeSelection.CfsSubsetEval -L |
| b - Search method | weka.attributeSelection.GreedyStepwise -C -B -N 71 |
| C - Selected model | weka.classifiers.trees.RandomForest -I 158 -K 1 -depth 8 |
| **Manual model selection** |  |
| 1 - Classification Via Regression | weka.classifiers.meta.ClassificationViaRegression -W weka.classifiers.trees.M5P -- -M 4.0 |
| 2 - Bayes Net | weka.classifiers.bayes.BayesNet -D -Q weka.classifiers.bayes.net.search.local.K2 -- -P 1 -S BAYES -E weka.classifiers.bayes.net.estimate.SimpleEstimator -- -A 0.5 |
| 3 - Naïve Bayes | weka.classifiers.bayes.NaiveBayes |
| Simple Logistic | weka.classifiers.functions.SimpleLogistic -I 0 -M 500 -H 50 -W 0.0 |
| LMT | weka.classifiers.trees.LMT -I -1 -M 15 -W 0.0 |
| Multi Class Classifier | weka.classifiers.meta.MultiClassClassifier -M 0 -R 2.0 -S 1 -W weka.classifiers.functions.Logistic -- -R 1.0E-8 -M -1 -num-decimal-places 4 |
| Logistic | weka.classifiers.functions.Logistic -R 1.0E-8 -M -1 -num-decimal-places 4 |
| Ada Boost M1 | weka.classifiers.meta.AdaBoostM1 -P 100 -S 1 -I 10 -W weka.classifiers.trees.DecisionStump |
| Random Sub Space | weka.classifiers.meta.RandomSubSpace -P 0.5 -S 1 -num-slots 1 -I 10 -W weka.classifiers.trees.REPTree -- -M 2 -V 0.001 -N 3 -S 1 -L -1 -I 0.0 |
| SGD | weka.classifiers.functions.SGD -F 0 -L 0.01 -R 1.0E-4 -E 500 -C 0.001 -S 1 |
| Random Committee | weka.classifiers.meta.RandomCommittee -S 1 -num-slots 1 -I 10 -W weka.classifiers.trees.RandomTree -- -K 0 -M 1.0 -V 0.001 -S 1 |
| Logit Boost | weka.classifiers.meta.LogitBoost -P 100 -L -1.7976931348623157E308 -H 1.0 -Z 3.0 -O 1 -E 1 -S 1 -I 10 -W weka.classifiers.trees.DecisionStump |
| SMO | weka.classifiers.functions.SMO -C 1.0 -L 0.001 -P 1.0E-12 -N 0 -V -1 -W 1 -K "weka.classifiers.functions.supportVector.PolyKernel -E 1.0 -C 250007" -calibrator "weka.classifiers.functions.Logistic -R 1.0E-8 -M -1 -num-decimal-places 4" |
| Iterative Classifier Optimized | weka.classifiers.meta.IterativeClassifierOptimizer -W weka.classifiers.meta.LogitBoost -L 50 -P 1 -E 1 -I 1 -F 10 -R 1 -percentage 0.0 -metric RMSE -S 1 -- -P 100 -L -1.7976931348623157E308 -H 1.0 -Z 3.0 -O 1 -E 1 -S 1 -I 10 -W weka.classifiers.trees.DecisionStump |
| Filtered Classifier | weka.classifiers.meta.FilteredClassifier -F "weka.filters.supervised.attribute.Discretize -R first-last -precision 6" -S 1 -W weka.classifiers.trees.J48 -- -C 0.25 -M 2 |
| PART | weka.classifiers.rules.PART -M 2 -C 0.25 -Q 1 |
| J48 | weka.classifiers.trees.J48 -C 0.25 -M 2 |
| Attribute Selected Classifier | weka.classifiers.meta.AttributeSelectedClassifier -E "weka.attributeSelection.CfsSubsetEval -P 1 -E 1" -S "weka.attributeSelection.BestFirst -D 1 -N 5" -W weka.classifiers.trees.J48 -- -C 0.25 -M 2 |
| REP Tree | weka.classifiers.trees.REPTree -M 2 -V 0.001 -N 3 -S 1 -L -1 -I 0.0 |
| Random Tree | weka.classifiers.trees.RandomTree -K 0 -M 1.0 -V 0.001 -S 1 |
| J Rip | weka.classifiers.rules.JRip -F 3 -N 2.0 -O 2 -S 1 |
| Decision Stump | weka.classifiers.trees.DecisionStump |
| One R | weka.classifiers.rules.OneR -B 6 |
|  |  |

^a^ Configuration was applied in order of A to C

Feature selection was conducted by either filter and wrapper method (built-in feature selection) in automatic model selection. But, only wrapper method was used in manual model selection. These procedures were applied after scheme-independent feature selection by association tests as described in main text. Our modelling, including the built-in feature selection and other complex configurations, can be reproduced by entering configuration code for each modelling (Table S1).

We optimized the best model from manual selection, which was Classification Via Regression (CVR). As described in the main text, the CVR were trained using three types of pulsatility index of uterine artery which were the right, mean, and lowest PI-UtA. These CVR models had different architectures (Multimedia Appendix 2). But, all of them consisted of decision tree with linear model (LM) in each leaf node. We used repeated 10-fold cross-validation, as described in the main text. Using only training subsets, we made threshold-accuracy curve in each subgroup that used each LM. We estimated the threshold in each that achieve the best accuracy in their own subgroup. Both point and interval estimates from 10 training subsets were plotted. The thresholds were used to predict validation subsets that were unobserved when estimating the thresholds. We used programming language R 3.6.1 in RStudio 1.2.1335 (RStudio Inc., Boston, MA, USA) to conduct these optimization procedures. The R notebook were shown for the CVR models with the right ([PDF](https://drive.google.com/open?id=1WfpO5CVvjS9UrVANu0EVz5mhbiToyI5P)), mean ([PDF](https://drive.google.com/open?id=1dMaCMLwHA4a-NE99efY7XSJ_WyoD0CDa)), and lowers ([PDF](https://drive.google.com/open?id=1QDg2FZg8pFFUTQy0NloySfJ-eJYVMTe0)) PI-UtA.
