# Supplementary material for: Prediction of Preeclampsia and Intrauterine Growth Restriction: Development of Machine Learning Models on a Prospective Cohort
Source: JMIR Med Inform. 2020 May 18;8(5):e15411. doi: 10.2196/15411 (PMC7265111; doi:10.2196/15411)
Supplement: Multimedia Appendix 2 [file medinform_v8i5e15411_app2.docx]

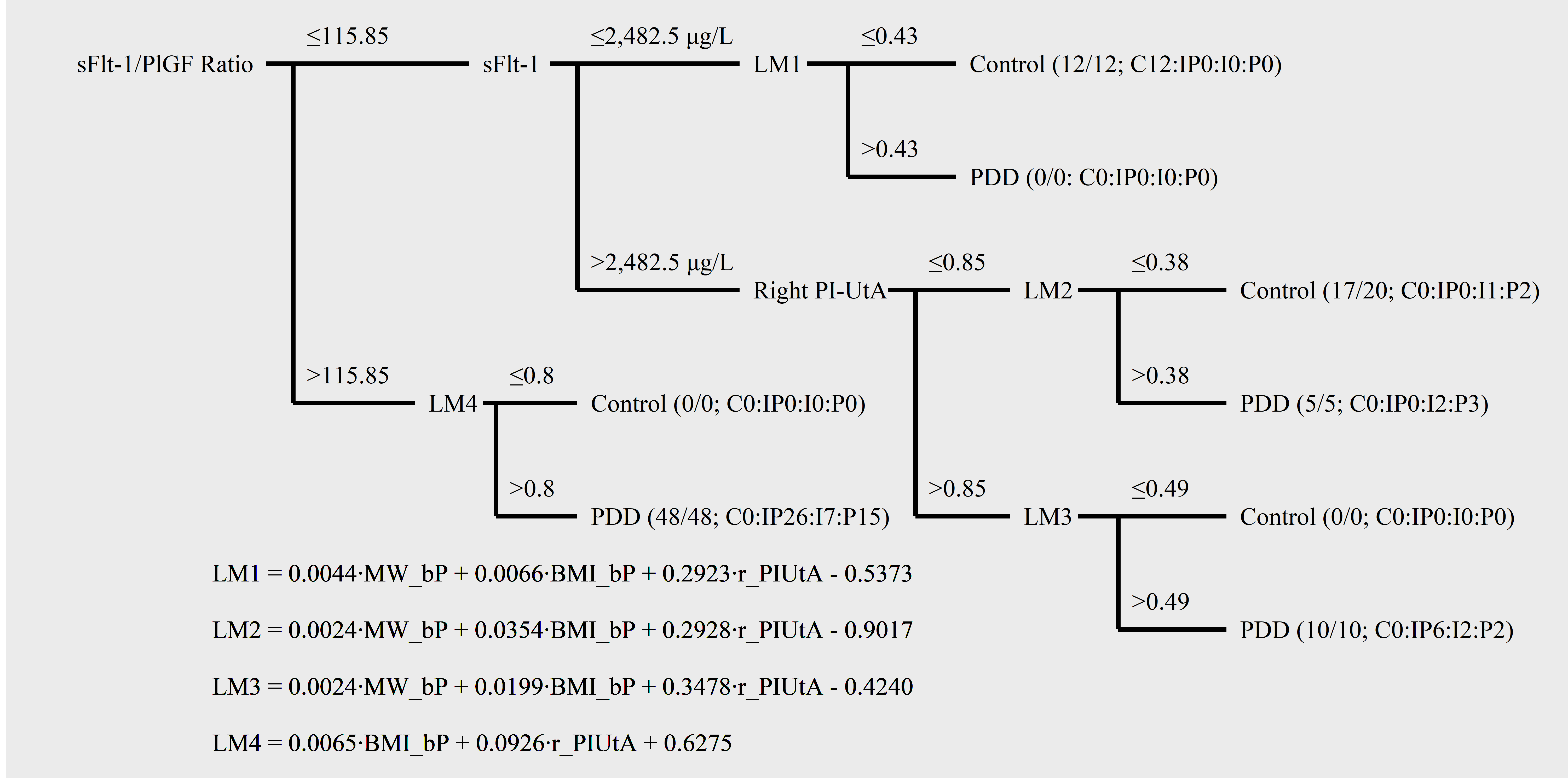
MULTIMEDIA APPENDIX 2

Characteristics of classification via regression (CVR) models with the right or mean of the pulsatility index of the uterine artery (PI-UtA)

# Prediction of preeclampsia and intrauterine growth restriction: development of machine learning models on a prospective cohort

Herdiantri Sufriyana^1,2^, MD, MSc; Yu-Wei Wu^1,3^, PhD; Emily Chia-Yu Su^1,3,4^, PhD

A


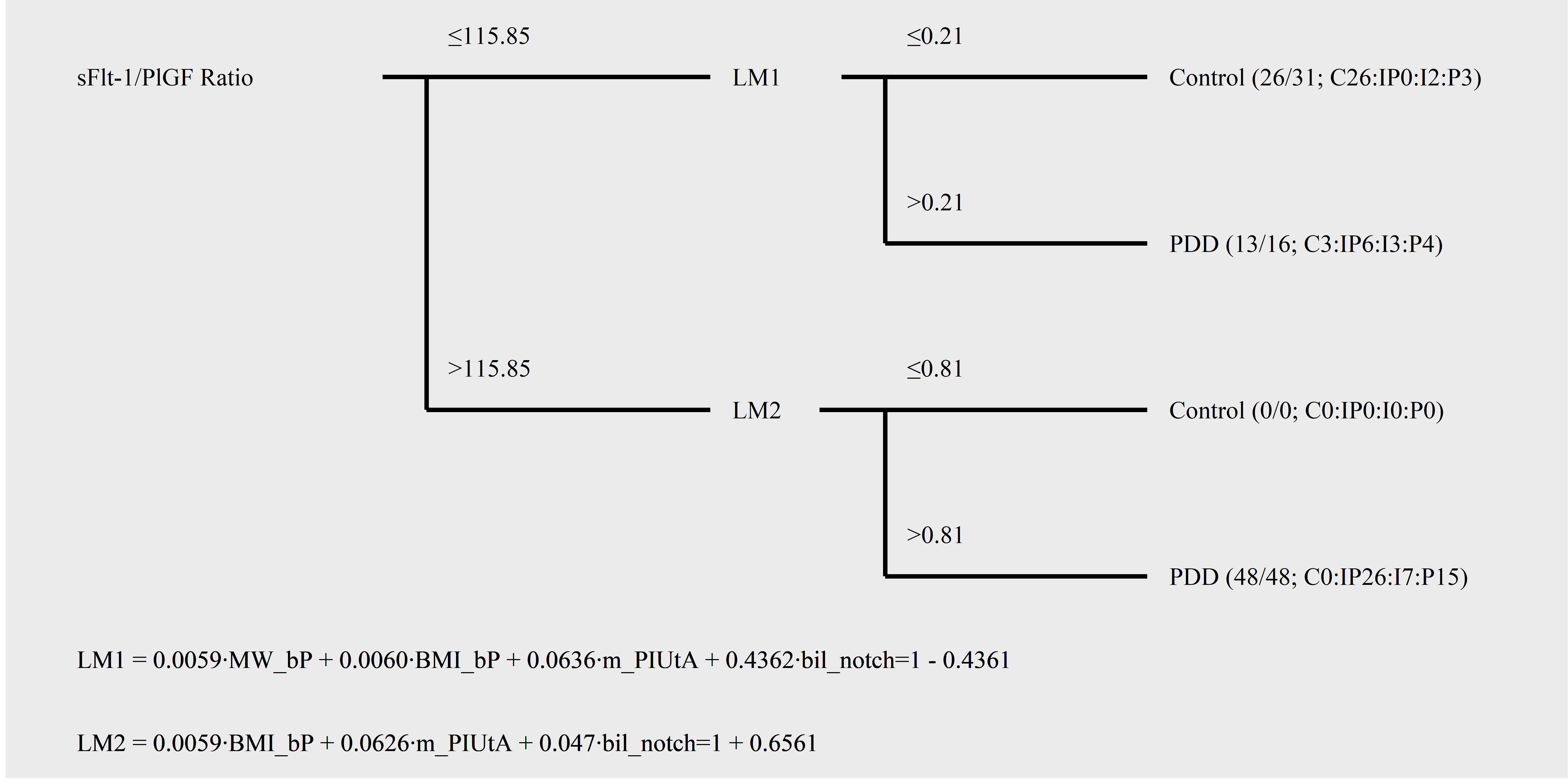
^1^Graduate Institute of Biomedical Informatics, College of Medical Science and Technology, Taipei Medical University, Taipei, Taiwan.

^2^Department of Medical Physiology, College of Medicine, University of Nahdlatul Ulama Surabaya, Surabaya, Indonesia.

^3^Clinical Big Data Research Center, Taipei Medical University Hospital, Taipei, Taiwan.

^4^Research Center for Artificial Intelligence in Medicine, Taipei Medical University, Taipei, Taiwan.

Instead of the mean PI-UtA, our Classification Via Regression (CVR) model chose the right PI-UtA (Figure S1 A). Then we developed a CVR model using the mean PI-UtA. This model shows different characteristics (Figure S1 B). Each linear model (LM) consisted of similar coefficients of maternal weight and BMI to either LM1 or LM4 from CVR with the right PI-UtA. It was also substituted with a combination of the mean PI-UtA and bilateral notch. A previous study has found no difference among the right, left and mean PI-UtA [1]. Bilateral notch had been shown improving predictive value of the mean PI-UtA [2]. In addition, each LM was chosen by 115.85 as cut-off for sFlt-1/PlGF ratio. It is exactly the same with other CVR models.

B

# References

Figure S1. Characteristics of Classification Via Regression model using the right (A) or the mean (B) PI-UtA. Fractions in leaf nodes consist of true predicted numbers (numerators) and all predicted ones (denominators). A ratio of true predicted numbers is shown for control (C), both IUGR and preeclampsia (IP), IUGR only (I), and preeclampsia only (P). PDD: placental dysfunction-related disorders. LM: linear model. MW_bP: maternal weight before pregnancy. BMI_bP: body mass index before pregnancy. r_PIUtA: the right pulsatility index of uterine artery. m_PIUtA: the mean pulsatility index of uterine artery. bil_notch=1: presence or absence of bilateral notch with value 0 or 1, respectively.

1. Ergin RN, Yayla M. Uterine artery pulsatility index and diastolic notch laterality according to the placental location. Clin Exp Obstet Gynecol 2015;42(5):640-643. PMID: [26524814](https://www.ncbi.nlm.nih.gov/pubmed/26524814)
2. Afrakhteh M, Moeini A, Taheri MS, Haghighatkhah HR, Fakhri M, Masoom N. Uterine Doppler velocimetry of the uterine arteries in the second and third trimesters for the prediction of gestational outcome. Rev Bras Ginecol Obstet 2014 Jan;36(1):35-39. PMID: [24554228](https://www.ncbi.nlm.nih.gov/pubmed/24554228)
