# Supplementary material for: Prediction of Preeclampsia and Intrauterine Growth Restriction: Development of Machine Learning Models on a Prospective Cohort
Source: JMIR Med Inform. 2020 May 18;8(5):e15411. doi: 10.2196/15411 (PMC7265111; doi:10.2196/15411)
Supplement: Multimedia Appendix 3 [file medinform_v8i5e15411_app3.docx]

MULTIMEDIA APPENDIX 3

Evaluation metrics and validation method for comparison with recent studies

# Prediction of preeclampsia and intrauterine growth restriction: development of machine learning models on a prospective cohort

Herdiantri Sufriyana^1,2^, MD, MSc; Yu-Wei Wu^1,3^, PhD; Emily Chia-Yu Su^1,3,4^, PhD

^1^Graduate Institute of Biomedical Informatics, College of Medical Science and Technology, Taipei Medical University, Taipei, Taiwan.

^2^Department of Medical Physiology, College of Medicine, University of Nahdlatul Ulama Surabaya, Surabaya, Indonesia.

^3^Clinical Big Data Research Center, Taipei Medical University Hospital, Taipei, Taiwan.

^4^Research Center for Artificial Intelligence in Medicine, Taipei Medical University, Taipei, Taiwan.

Table S1. Predictive performances shown by the models in this study as well as validation method compared with those from recent studies.

| Sources* | PPV (%) | ACC(%) | MCC | Validation method |
| --- | --- | --- | --- | --- |
| **This study** |  |  |  |  |
| CVR1 (right PI-UtA) | 98.3 (94.9-100) | 92.6 (86.3-98.9) | 0.84 (0.71-0.98) | Repeated 10-folds cross-validation |
| CVR2 (mean PI-UtA) | 100 (100-100) | 96.8 (93.7-100) | 0.93 (0.87-1.00) | Repeated 10-folds cross-validation |
| CVR3 (lowest PI-UtA) | 100 (100-100) | 96.8 (93.7-100) | 0.93 (0.87-1.00) | Repeated 10-folds cross-validation |
| 158-trees Random Forest | 99.2 (98.4-100) | 96.3 (91.4-100) | 0.93 (0.82-1.00) | Repeated 10-folds cross-validation |
| **Recent studies** |  |  |  |  |
| Wright A *et al.* (2019) | 0.9 (0.8-1.0) | 89.9 (89.6-90.2) | 0.08 (0.07-0.09) | Markov chain Monte Carlo cross-validation; Independent test set |
| Wright D *et al.* (2019) | 53.1 (50-56.3) | 90.0 (88.7-91.2) | 0.64 (0.57-0.71) | 5-folds cross-validation |
| Tan MY *et al.* (2018) | 23.8 (22.1-25.6) | 90.0 (89.6-90.2) | 0.43 (0.39-0.47) | Markov chain Monte Carlo cross-validation |
| Sonek J *et al.* (2018) | 17.7 | 94.9 | 0.37 | Stratified shuffle split cross-validation [5];  Independent test set |
| Perales A *et al.* (2017) | 31.7 | 94.6 | 0.49 | Whole training set |
| Nuriyeva G *et al.* (2017) | 20.2 | 89.8 | 0.36 | Test-split cross-validation |
| O'Gorman N *et al.* (2017) | 1.8 (1.6-2.0) | 90.0 (90.0-90.0) | 0.12 (0.11-0.13) | Stratified shuffle split cross-validation |
| Gallo DM *et al.* (2016) | 5.3 (4.7-5.9) | 90.0 (90.0-90.0) | 0.2 (0.17-0.22) | 5-folds cross-validation |
| Tsiakkas A *et al.* (2016) | 5.5 (5.3-5.8) | 90.04 (90.01-90.07) | 0.22 (0.21-0.23) | 10-folds cross-validation |
| Andrietti S *et al.* (2016) | 11.4 (10.0-12.7) | 89.9 (89.7-90.4) | 0.28 (0.24-0.32) | 5-folds cross-validation |
| O'Gorman N *et al.* (2016) | 1.6 (1.5-1.8) | 90.0 (90.0-90.0) | 0.11 (0.1-0.12) | Stratified shuffle split cross-validation |
| Wright D *et al.* (2015) | 1.7 (1.5-1.9) | 90.14 (90.12-90.16) | 0.1 (0.08-0.11) | 5-folds cross-validation |

| $PPV (\%)=\frac{TP}{TP+FP}\times100\%$ | $ACC \left( \% \right)=\frac{TP+TN}{TP+FP+TN+FN}\times100\%$ | $TP=P\times Sensitivity \left( \% \right)$ | $TN=N\times Specificity (\%)$ |
| --- | --- | --- | --- |
| $MCC=\frac{TP\times TN-FN\times FP}{\sqrt{(TP+FN)(TP+FP)(TN+FP)(TN+FN)}}$ | | $FN=P-TP$ | $FP=N-TN$ |

# Abbreviations

CVR: Classification Via Regression

ACC: accuracy

FN: false negatives

FP: false positives

MCC: Matthew’s correlation coefficient

N: negatives

PPV: positive predictive value

P: positives

TN: true negatives

TP: true positives

# References

1. Wright A, Wright D, Syngelaki A, Georgantis A, Nicolaides KH. Two-stage screening for preterm preeclampsia at 11–13 weeks’ gestation. Am J Obstet Gynecol 2019;220(2):197.e191-197.e111. PMID: [30414394](https://www.ncbi.nlm.nih.gov/pubmed/30414394)
2. Wright D, Tan MY, O'Gorman N, Poon LC, Syngelaki A, Wright A, Nicolaides KH. Predictive performance of the competing risk model in screening for preeclampsia. Am J Obstet Gynecol 2019;220(2):199.e191-199.e113. PMID: [30447210](https://www.ncbi.nlm.nih.gov/pubmed/30447210)
3. Tan MY, Wright D, Syngelaki A, Akolekar R, Cicero S, Janga D, Singh M, Greco E, Wright A, Maclagan K, Poon LC, Nicolaides KH. Comparison of diagnostic accuracy of early screening for pre-eclampsia by NICE guidelines and a method combining maternal factors and biomarkers: results of SPREE. Ultrasound Obstet Gynecol 2018 Jun;51(6):743-750. PMID: [29536574](https://www.ncbi.nlm.nih.gov/pubmed/29536574)
4. Sonek J, Krantz D, Carmichael J, Downing C, Jessup K, Haidar Z, Ho S, Hallahan T, Kliman HJ, McKenna D. First-trimester screening for early and late preeclampsia using maternal characteristics, biomarkers, and estimated placental volume. Am J Obstet Gynecol 2018;218(1):126.e121-126.e113. PMID: [29097177](https://www.ncbi.nlm.nih.gov/pubmed/29097177)
5. O'Gorman N, Wright D, Syngelaki A, Akolekar R, Wright A, Poon LC, Nicolaides KH. Competing risks model in screening for preeclampsia by maternal factors and biomarkers at 11-13 weeks gestation. Am J Obstet Gynecol 2016 Jan;214(1):103.e101-103.e112. PMID: [26297382](https://www.ncbi.nlm.nih.gov/pubmed/26297382)
6. Perales A, Delgado JL, de la Calle M, García-Hernández JA, Escudero AI, Campillos JM, Sarabia MD, Laíz B, Duque M, Navarro M, Calmarza P, Hund M, Álvarez FV, Romero A, Cabrera F, Vázquez M, Moreno F, Vaquerizo Ó, Miguel M, de Paco C, Pertegal M, Arteaga A, Jesús Franco M, Envid B, Martin E, Bartha JL, Buño A, Pérez G, Roig S, Gómez R, Hervás D, de la Cruz ÁA, López N, Melero V, Calero M, de Ramón M, Paya A. sFlt-1/PlGF for prediction of early-onset pre-eclampsia: STEPS (Study of Early Pre-eclampsia in Spain). Ultrasound Obstet Gynecol 2017;50(3):373-382. PMID: [27883242](https://www.ncbi.nlm.nih.gov/pubmed/27883242)
7. Nuriyeva G, Kose S, Tuna G, Kant M, Akis M, Altunyurt S, Islekel GH, Dogan OE. A prospective study on first trimester prediction of ischemic placental diseases. Prenat Diagn 2017 Apr;37(4):341-349. PMID: [28165141](https://www.ncbi.nlm.nih.gov/pubmed/28165141)
8. O'Gorman N, Wright D, Poon LC, Rolnik DL, Syngelaki A, Wright A, Akolekar R, Cicero S, Janga D, Jani J, Molina FS, de Paco Matallana C, Papantoniou N, Persico N, Plasencia W, Singh M, Nicolaides KH. Accuracy of competing-risks model in screening for pre-eclampsia by maternal factors and biomarkers at 11-13 weeks' gestation. Ultrasound Obstet Gynecol 2017 Jun;49(6):751-755. PMID: [28067011](https://www.ncbi.nlm.nih.gov/pubmed/28067011)
9. Gallo DM, Wright D, Casanova C, Campanero M, Nicolaides KH. Competing risks model in screening for preeclampsia by maternal factors and biomarkers at 19-24 weeks' gestation. Am J Obstet Gynecol 2016 May;214(5):619.e611-619.e617. PMID: [26627730](https://www.ncbi.nlm.nih.gov/pubmed/26627730)
10. Tsiakkas A, Saiid Y, Wright A, Wright D, Nicolaides KH. Competing risks model in screening for preeclampsia by maternal factors and biomarkers at 30-34 weeks' gestation. Am J Obstet Gynecol 2016 Jul;215(1):87.e81-87.e17. PMID: [26875953](https://www.ncbi.nlm.nih.gov/pubmed/26875953)
11. Andrietti S, Silva M, Wright A, Wright D, Nicolaides KH. Competing-risks model in screening for pre-eclampsia by maternal factors and biomarkers at 35-37 weeks' gestation. Ultrasound Obstet Gynecol 2016 Jul;48(1):72-79. PMID: [26566592](https://www.ncbi.nlm.nih.gov/pubmed/26566592)
12. Wright D, Syngelaki A, Akolekar R, Poon LC, Nicolaides KH. Competing risks model in screening for preeclampsia by maternal characteristics and medical history. Am J Obstet Gynecol 2015 Jul;213(1):62.e61-62.e10. PMID: [25724400](https://www.ncbi.nlm.nih.gov/pubmed/25724400)
