# Supplementary material for: Prediction of Preeclampsia and Intrauterine Growth Restriction: Development of Machine Learning Models on a Prospective Cohort
Source: JMIR Med Inform. 2020 May 18;8(5):e15411. doi: 10.2196/15411 (PMC7265111; doi:10.2196/15411)
Supplement: Multimedia Appendix 4 [file medinform_v8i5e15411_app4.pdf]

Click Here to visit the interactive model

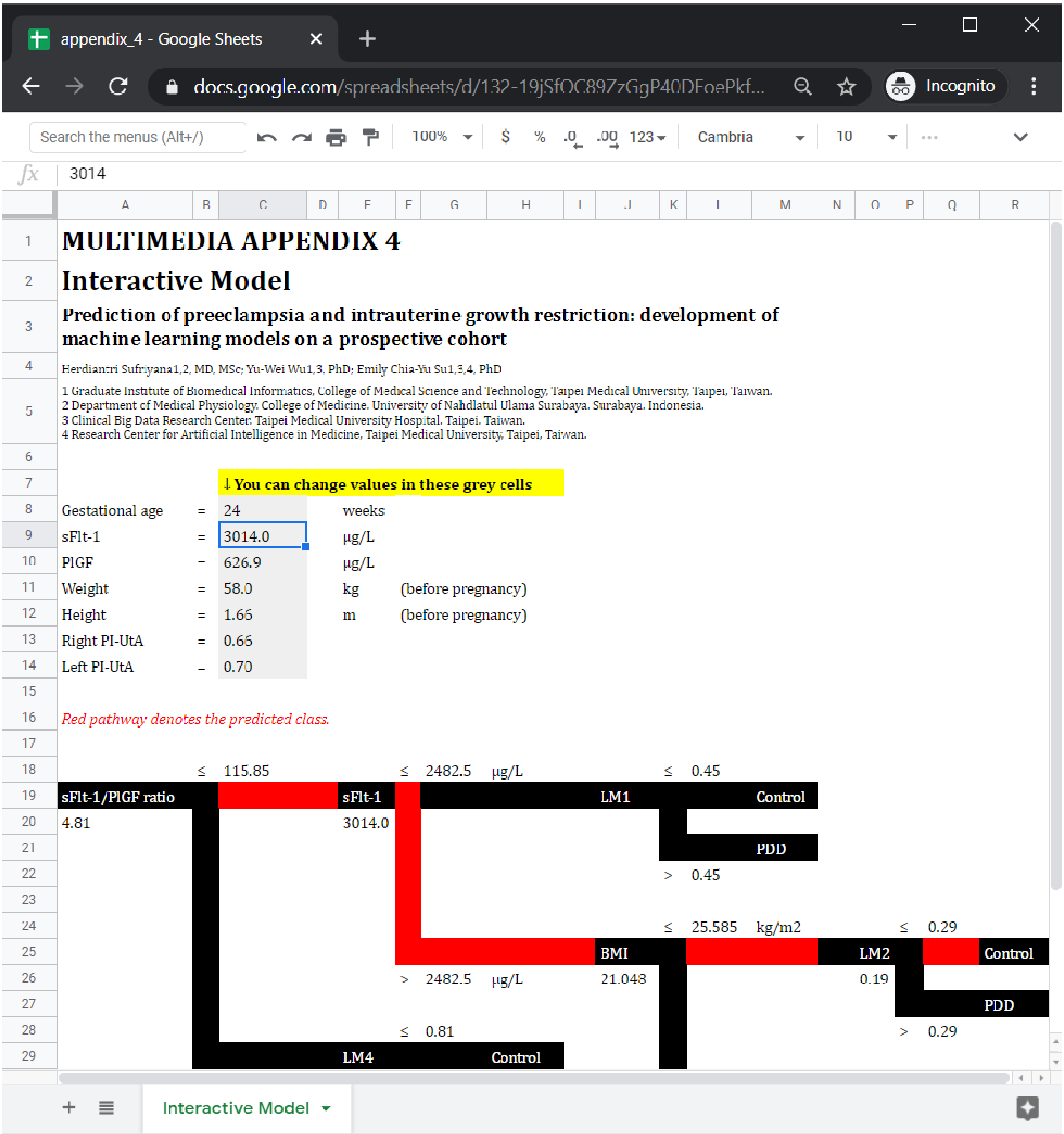

MULTIMEDIA APPENDIX 4

Interactive Model

Prediction of preeclampsia and intrauterine growth restriction: development of machine learning models on a prospective cohort

Herdiantri Sufriyana1,2, MD, MSc; Yu-Wei Wu1,3, PhD; Emily Chia-Yu Su1,3,4, PhD

1 Graduate Institute of Biomedical Informatics, College of Medical Science and Technology, Taipei Medical University, Taipei, Taiwan.
2 Department of Medical Physiology, College of Medicine, University of Nahdlatul Ulama Surabaya, Surabaya, Indonesia.
3 Clinical Big Data Research Center, Taipei Medical University Hospital, Taipei, Taiwan.
4 Research Center for Artificial Intelligence in Medicine, Taipei Medical University, Taipei, Taiwan.

↓You can change values in these grey cells

Gestational age	=	24	weeks	
sFlt-1	=	3014.0	µg/L	
PlGF	=	626.9	µg/L	
Weight	=	58.0	kg	(before pregnancy)
Height	=	1.66	m	(before pregnancy)
Right PI-UtA	=	0.66		
Left PI-UtA	=	0.70		

Red pathway denotes the predicted class.
